# Palmitoylation of the Cysteine Residue in the DHHC Motif of a Palmitoyl Transferase Mediates Ca^2+^ Homeostasis in *Aspergillus*

**DOI:** 10.1371/journal.pgen.1005977

**Published:** 2016-04-08

**Authors:** Yuanwei Zhang, Qingqing Zheng, Congcong Sun, Jinxing Song, Lina Gao, Shizhu Zhang, Alberto Muñoz, Nick D. Read, Ling Lu

**Affiliations:** 1 Jiangsu Key Laboratory for Microbes and Functional Genomics, Jiangsu Engineering and Technology Research Center for Microbiology; College of Life Sciences, Nanjing Normal University, Nanjing, China; 2 Manchester Fungal Infection Group, Institute of Inflammation and Repair, University of Manchester, Manchester, United Kingdom; University of Nebraska-Lincoln, UNITED STATES

## Abstract

Finely tuned changes in cytosolic free calcium ([Ca^2+^]_c_) mediate numerous intracellular functions resulting in the activation or inactivation of a series of target proteins. Palmitoylation is a reversible post-translational modification involved in membrane protein trafficking between membranes and in their functional modulation. However, studies on the relationship between palmitoylation and calcium signaling have been limited. Here, we demonstrate that the yeast palmitoyl transferase *Sc*Akr1p homolog, AkrA in *Aspergillus nidulans*, regulates [Ca^2+^]_c_ homeostasis. Deletion of *akrA* showed marked defects in hyphal growth and conidiation under low calcium conditions which were similar to the effects of deleting components of the high-affinity calcium uptake system (HACS). The [Ca^2+^]_c_ dynamics in living cells expressing the calcium reporter aequorin in different *akrA* mutant backgrounds were defective in their [Ca^2+^]_c_ responses to high extracellular Ca^2+^ stress or drugs that cause ER or plasma membrane stress. All of these effects on the [Ca^2+^]_c_ responses mediated by AkrA were closely associated with the cysteine residue of the AkrA DHHC motif, which is required for palmitoylation by AkrA. Using the acyl-biotin exchange chemistry assay combined with proteomic mass spectrometry, we identified protein substrates palmitoylated by AkrA including two new putative P-type ATPases (Pmc1 and Spf1 homologs), a putative proton V-type proton ATPase (Vma5 homolog) and three putative proteins in *A*. *nidulans*, the transcripts of which have previously been shown to be induced by extracellular calcium stress in a CrzA-dependent manner. Thus, our findings provide strong evidence that the AkrA protein regulates [Ca^2+^]_c_ homeostasis by palmitoylating these protein candidates and give new insights the role of palmitoylation in the regulation of calcium-mediated responses to extracellular, ER or plasma membrane stress.

## Author Summary

Palmitoylation is a reversible post-translational modification catalyzed by palmitoyl acyltransferases (PATs) and proteins that undergo this modification are involved in numerous intracellular functions. Yeast Akr1p was the first characterized PAT whilst HIP14, an Akr1p homolog in human, is one of the most highly conserved of 23 human PATs that catalyze the addition of palmitate to the Huntington protein which is of major importance in Huntington’s disease. Calcium serves numerous signaling and structural functions in all eukaryotes. However, studies on the relationship between calcium signaling and palmitoylation are lacking. In this study, we demonstrate that the palmitoyl transferase Akr1 homolog in the filamentous fungus *Aspergillus nidulans*, similar to the high-affinity calcium uptake system (HACS), is required for normal growth and sporulation in the presence of low extracellular calcium. We find that AkrA dysfunction decreases the transient increase in cytosolic free calcium induced by a high extracellular calcium stress, tunicamycin (which induces endoplasmic reticulum stress) or the antifungal agent itraconazole (which induces plasma membrane stress). The influence of AkrA on all of these processes involves its DHHC motif, which is required for palmitoylation of various proteins associated with many processes including calcium signaling and membrane trafficking. Our findings provide evidence for a crucial link between calcium signaling and palmitoylation, suggesting a possible role in the mechanistic basis of human PAT-related diseases. These results also indicate that regulators of posttranslational modification may provide promising antifungal targets for new therapies.

## Introduction

In all eukaryotic cells, the cytosolic free calcium ([Ca^2+^]_c_) concentration is strictly and precisely controlled by complex interactions between various calcium-channels, calcium-pumps and calcium-antiporters and by calcium buffering in the cytoplasm. Finely tuned changes in [Ca^2+^]_c_ mediate a variety of intracellular functions, and disruption of [Ca^2+^]_c_ homeostasis can lead to various pathological conditions [1]. In fungi, numerous studies have shown that calcium signaling is involved in regulating a wide range of processes including cell morphogenesis, cell cycle progression, stress responses and virulence [2]. Two different calcium uptake systems in the plasma membrane have been identified in most fungal species: the high-affinity Ca^2+^ influx system (HACS) and the low-affinity calcium influx system (LACS) [3–5]. The main components of the HACS are primarily composed of an α-subunit of the mammalian voltage-gated Ca^2+^-channel homolog Cch1 and a stretch-activated β-subunit called Mid1. Loss of the HACS results in an inability to grow under low-calcium conditions. In addition, fungi possess a range of other calcium P-type ATPases and calcium transporters that play important roles in calcium signaling and homeostasis [6]. Upon stimulation, calcium is rapidly taken up from the extracellular environment or released from these intracellular calcium stores and either interacts with the primary intracellular calcium sensor/receptor calmodulin or directly regulates that activity of other proteins. When the calcium signal binds to calmodulin this results in a conformational change in the protein allowing it to interact with and regulate the activity of various target proteins involved in converting the original stimuli into cellular responses. The [Ca^2+^]_c_ increase is transient because various calcium-pumps and calcium-antiporters, as well as the cytoplasmic calcium buffering, subsequently return the [Ca^2+^]_c_ to its normally low resting level within the cytosol [7,8].

The phosphatase calcineurin is an important [Ca^2+^]_c_ transient effector and is conserved from yeast to humans. Its most well known target in fungi is the transcription factor Crz1 (calcineurin responsive zinc finger 1) [9,10]. In vegetatively growing *S*. *cerevisiae* cells, [Ca^2+^]_c_ concentrations are normally maintained at low non-signaling levels. During this stage, Crz1 is fully phosphorylated, localized to the cytoplasm, and transcriptionally inactive [11,12]. When fungal cells are exposed to chemicals that induce plasma membrane stress (*e*.*g*. by azole antifungals) or endoplasmic reticulum (ER) stress (*e*.*g*. by tunicamycin), or are under low calcium conditions, the HACS is activated. These stimuli result in calcium uptake and a transient increase in [Ca^2+^]_c_ which leads to calcineurin activation and subsequent Crz1 de-phosphorylation. Crz1 is then recruited to nuclei where it transcriptionally regulates downstream signaling pathways to alleviate cellular stress and promote cell survival [13,14]. Interestingly, there are no known mammalian Crz1 orthologs, but mammals express another calcineurin sensitive transcription factor target, known as NFAT (nuclear factor of activated T-cells). Crz1 does not belong to the NFAT family, but the Zn-finger domains in Crz1 and NFAT bind specific DNA sequences within the promoter regions of calcineurin-dependent response elements (CDREs) to activate transcription [15,16]. In the filamentous fungus *Aspergillus nidulans*, there is a calcineurin-dependent Crz1 homolog, known as CrzA. Interestingly, calcineurin deletion causes more severe growth defects than CrzA deletion in this species, suggesting that calcineurin has additional target proteins other than CrzA [17,18].

Palmitoylation is a reversible posttranslational modification that catalyzes the attachment of palmitate to cytoplasmic cysteine residues of soluble and transmembrane proteins. Palmitoyl transferases (PATs) are known to be responsible for palmitoylation. The defining feature of PATs is the presence of a cysteine-rich domain (CRD) with an Asp-His-His-Cys (DHHC) motif, which is required for PAT activity. Many proteins that require palmitoylation are involved in cellular signaling, membrane trafficking and synaptic transmission [19–21]. There are more than 20 encoded DHHC proteins in mammalian genomes, and there is now a major effort to verify DHHC-substrate partners and determine how their interaction specificity is encoded [22]. Several lines of recent evidence have shown that protein palmitoylation influences various cell functions, physiology and pathophysiology [23–25].

In this study, we have demonstrated that AnAkrA in *A*. *nidulans* and AfAkrA in *A*. *fumigatus*, which are homologs of the yeast palmitoyl transferase ScAkr1p, have similar function to the HACS in the presence of low extracellular calcium. The *akrA* deletion resulted in marked defects in hyphal extension and conidiation, especially under low calcium conditions. Moreover, using codon-optimized aequorin as a calcium reporter in living cells, we found that AkrA dysfunction significantly decreased the amplitude of the [Ca^2+^]_c_ transient induced by an extracellular calcium stimulus, ER stress caused by tunicamycin or plasma membrane stress resulting from itraconazole, respectively. Our data suggest that these [Ca^2+^]_c_ responses are mediated by the palmitoylation of the cysteine residue of the DHHC motif in AkrA. Moreover, we have identified that two new putative P-type ATPases (Pmc1 and Spf1 homologs), a putative proton V-type proton ATPase (Vma5 homolog) and three putative CrzA-dependent proteins, are palmitoylated substrates of the AkrA protein. To our knowledge, this is the first report that a palmitoylation protein is involved in regulating eukaryotic calcium signaling.

## Results

### Phenotypic characterization of the Golgi-localized AkrA

**Fig 1:**
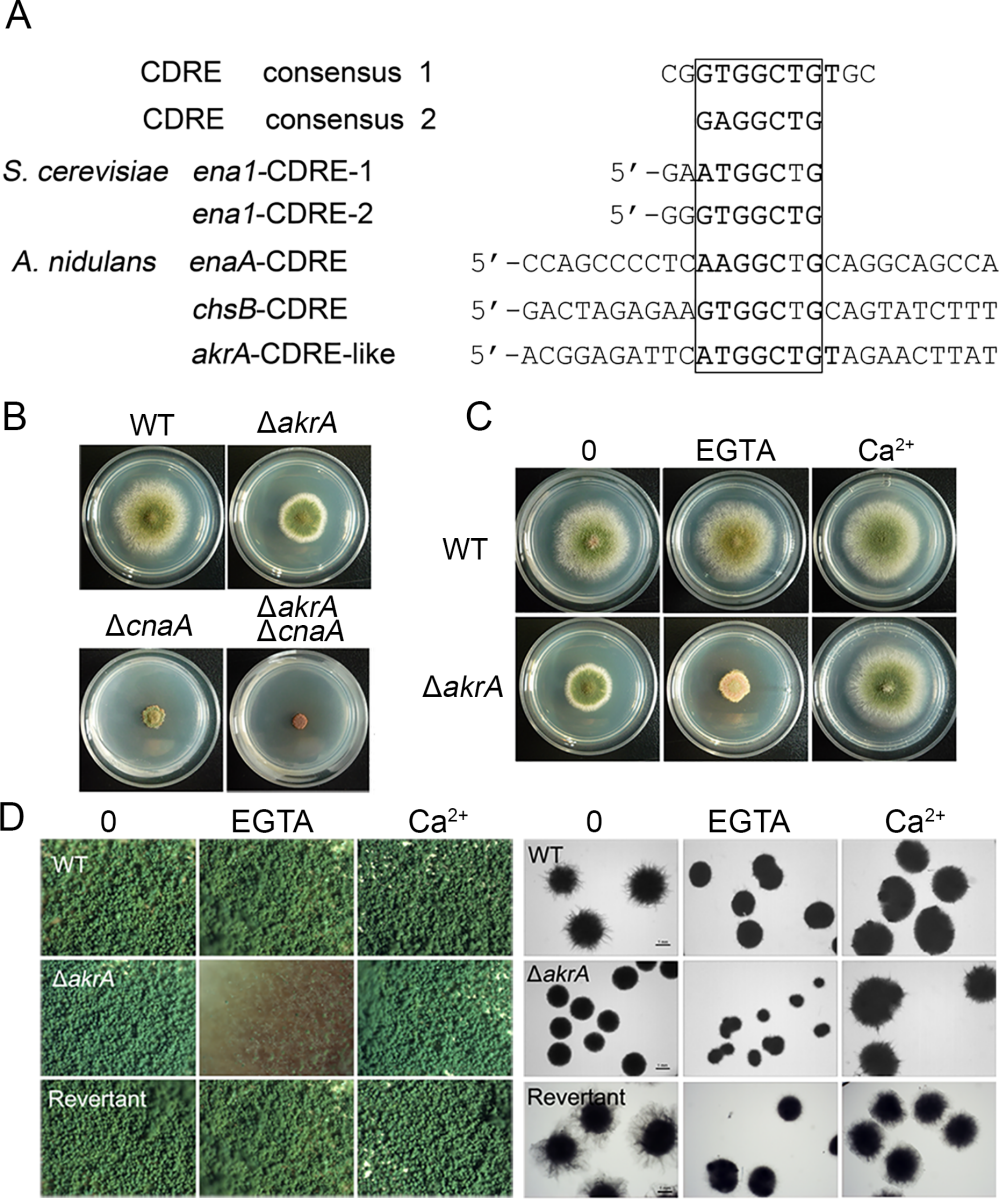
Identification of AkrA in *A*. *nidulans*. A. Alignment of Crz1/CrzA DNA-binding sites. CDRE consensus sequences 1 and 2 correspond to those described in previous studies. A CDRE-like sequence was identified at 398 bp (*akrA*, AN5824.4) upstream of its respective start codon. B. The colony morphologies of TN02A7 (WT), Δ*akrA*, Δ*cnaA* and Δ*akrA*Δ*cnaA* strains grown on minimal media at 37°C for 2.5 days. C. The TN02A7 (WT) and Δ*akrA* strains were incubated at 37°C for 2.5 days on minimal medium in the presence or absence of 1 mM EGTA or 20 mM CaCl_2_. D. The pattern of conidiation and hyphal branching in TN02A7 (WT), Δ*akrA* and revertant strains. Images were taken with a stereo microscope after culturing colonies for 2.5 days on solid non-inducing medium and culturing mycelial balls for 24 h in liquid non-inducing medium, respectively.

Based on a NCBI BLASTp search (http://www.ncbi.nlm.nih.gov/BLAST/), we identified a putative ortholog of NFAT in *A*. *nidulans*, AkrA (AN5824.4, Accession: XP_663428.1), which encodes a putative palmitoyltransferase. However, it showed low identity (less than 20%) or similarity (less than 30%) to mammalian NFAT based on full-length sequences. Interestingly, a bioinformatic analysis revealed that the promoter region contains a putative calcineurin-dependent-response-element (CDRE-like) motif. As shown in Fig 1A, we identified a CDRE-like sequence at 398 bp (*akrA*, AN5824.4), upstream of this gene’s start codon [26,27]. These data suggest that AkrA may be a component of the calcium signaling machinery. To further explore the function of the *akrA* gene and its relationship to calcineurin, the full-length deletion strain was constructed by homologous gene replacement employing a self-excising recyclable cassette that contains an *AfpyrG* gene as a selectable marker. Diagnostic PCR analysis of the resulting strain Δ*akrA* confirmed the homologous replacement (S1A Fig). We also generated Δ*akrA*Δ*cnaA* double mutants through genetic crosses (the *cnaA* gene encodes the catalytic subunit of calcineurin). The Δ*akrA* mutant produced smaller colonies compared to that of the parental wild-type strain, when grown on minimal medium. In comparison, the Δ*cnaA* mutant exhibited severe growth defects on minimal medium. Moreover, the double mutant had a smaller colony size and underwent less conidiation than the single mutants (Fig 1B). These results suggest that *akrA* and *cnaA* may have different functions in *A*. *nidulans*. Therefore, the double deletion mutant exacerbates the growth defects on minimal medium.

We next tested whether low external calcium conditions could affect the colony phenotype in the *akrA* deletion mutant. When conidia were spot inoculated onto the solid minimal medium containing the calcium chelator EGTA and were allowed to grow at 37°C for 2.5 days, the Δ*akrA* mutant exhibited increased EGTA sensitivity compared to the parental wild-type strain. As shown in Fig 1C, the *akrA* deletion exhibited markedly reduced conidial formation and colony growth under low-calcium conditions. Since, mutants of the HACS components have been previously shown to exhibit similar defects under low calcium conditions [28–30], we next examined whether AkrA was a potential novel HACS component. To determine whether the defects in the Δ*akrA* mutant could be rescued by high extracellular calcium, we inoculated Δ*akrA* mutant conidia on minimal medium supplemented with 20 mM Ca^2+^. We found that the colony diameter of the Δ*akrA* mutant was restored almost to the same diameter of the parental wild-type strain by the addition of extracellular calcium (Fig 1C), indicating that exogenous calcium could completely rescue the colony growth defect caused by AkrA loss. We further examined conidiation in the Δ*akrA* mutant in a calcium-limited environment (*i*.*e*. in the presence of EGTA) with a stereomicroscope (Fig 1D left panels). The results showed that the vegetative mycelia from the parental wild-type strain were capable of producing numerous conidia under low-calcium conditions. In contrast, conidiation was almost completely abolished in the Δ*akrA* mutant on minimal media supplemented with EGTA (1 mM) (Fig 1D left panels). In submerged liquid culture, the wild-type strain displayed robust polarized hyphal growth around the margins of mycelial balls, whereas the Δ*akrA* mutant showed smooth margins around small mycelial balls (Fig 1D right panels). Consistently, the Δ*akrA* mutant had a significantly reduced biomass, germination rate, and colony size compared to the parental strain on minimal media (S3 Fig). Moreover, ectopically expressed *akrA* was able to completely rescue these defects in the *akrA* deletion strain (Fig 1D), establishing that these phenotypes were specific to the loss of *akrA*. In addition, we deleted the *akrA* homolog gene in *A*. *fumigatus*. Similar to the Δ*akrA* phenotypes in *A*. *nidulans*, the Δ*AfakrA* mutant displayed hypersensitivity to the low calcium conditions, and its phenotypic defects could be rescued by high extracellular calcium (S2 Fig). Thus, these data are consistent with AkrA being involved in calcium uptake especially in a calcium-limited environment.

**Fig 2:**
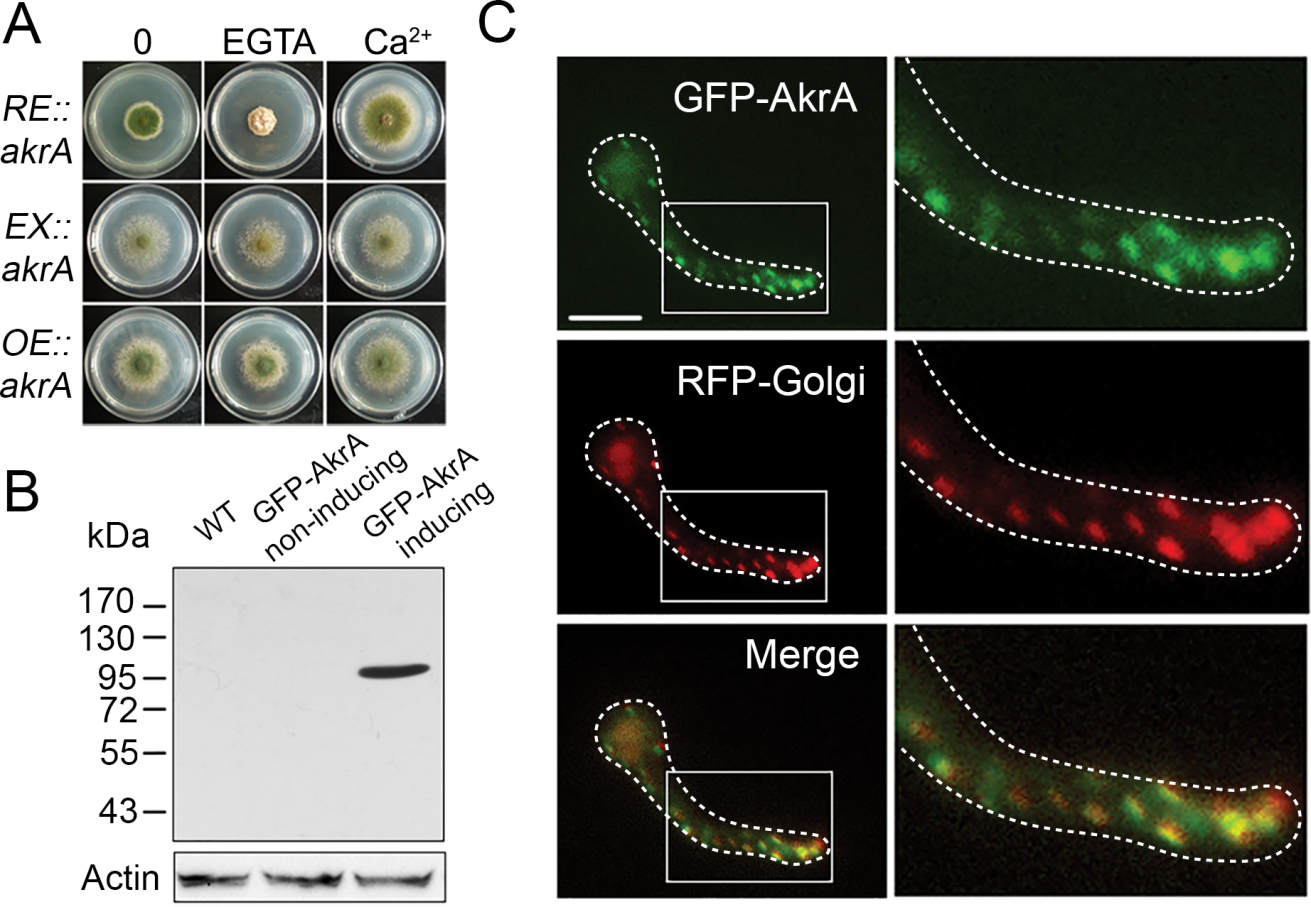
Phenotypic characterization of Golgi-localized AkrA. A. The phenotypic characterization of *akrA* under control of the *alcA(p)* conditional promoter. The colony images show corresponding strains grown on the non-inducing medium (*RE*::*akrA*), inducing medium (*EX*::*akrA*) and overexpressing medium (*OE*::*akrA*) at 37°C for 2.5 days. B. Western blot analysis indicated a fusion protein of GFP-AkrA was detected with a predicted size of approximately 100 kDa by using an anti-GFP antibody. GFP-AkrA non-inducing and GFP-AkrA inducing represent *alcA(p)*::GFP*-akrA* grown in liquid non-inducing medium and inducing medium, respectively. Anti-actin antibody against actin was used as an internal control of loading. C. Colocalization of GFP-AkrA and the GEs marker mRFP-PH^OSBP^. A strain carrying transgenes expressing the two fluorescent reporters was imaged using GFP and mRFP specific filter sets. The yellow color in the merged image shows the co-localization. Bar, 5 μm.

To further confirm and assess the localization and the molecular mass of AkrA, we generated a conditional expression allele, *alcA(p)*::GFP*-akrA*, referred to here as ZYA09 (S1B Fig). In this conditional allele, *akrA* expression was assumed to be regulated by the carbon source, as it was not induced by glucose, induced by glycerol, and overexpressed to high levels by L-threonine [31]. To determine whether this conditional allele behaved as predicted, we inoculated the ZYA09 strain in liquid media for 18 h, which promoted induction, non-induction or overexpression. As expected, the *akrA* mRNA level was approximately 20-fold higher when grown in overexpressing medium compared to that grown in non-inducing medium, which was 12-fold higher than that in inducing medium (S4B Fig). Moreover, the conditional strain ZYA09 displayed an identical phenotype to the parental wild-type strain when grown on the inducing or the overexpressing media, indicating that the fusion GFP-AkrA protein was functional and that the assumed *akrA* over-expression had no detectable effects in *A*. *nidulans*. In comparison, when grown on the non-inducing medium, the conditional allele *alcA(p)*::GFP*-akrA* exhibited an identical phenotype to the Δ*akrA* mutant, confirming a consistent phenotype for the loss of AkrA and for the knock-down of AkrA (Figs 2A and 1C). Western blotting showed a band at approximately 110 kDa in the GFP-AkrA strain grown under inducing or overexpressing conditions using an anti-GFP antibody but no such a band appeared in the parental wild-type strain or the conditional allele (ZYA09) under the non-inducing condition (Fig 2B). These results indicate that the molecular mass of AkrA is approximately 80 kDa because GFP is a 27 kDa protein.

Microscopic examination showed that the AkrA-GFP localization pattern resembled that of the Golgi previously reported in *A*. *nidulans* [32]. To confirm this we generated the strain ZYA13 by genetically crossing the *alcA(p)*::GFP*-akrA* strain ZYA09 with the MAD2013 strain in which the late Golgi marker (*gpdA*^*mini*^::*mRFP-PH*^*OSBP*^), consisting of the pleckstrin homology domain of the human oxysterol binding protein (PH^OSBP^) fused to mRFP was included [33,34]. Spores of the ZYA13 strain were incubated in non-inducing medium at 37°C for 10 h and were then shifted to the overexpression medium for 6 h. Microscopic examination of the young germlings produced under these conditions showed the majority of GFP-AkrA proteins colocalized with mRFP-PH^OSBP^ late Golgi marker (Fig 2C).

### The DHHC motif is required for AkrA function

**Fig 3:**
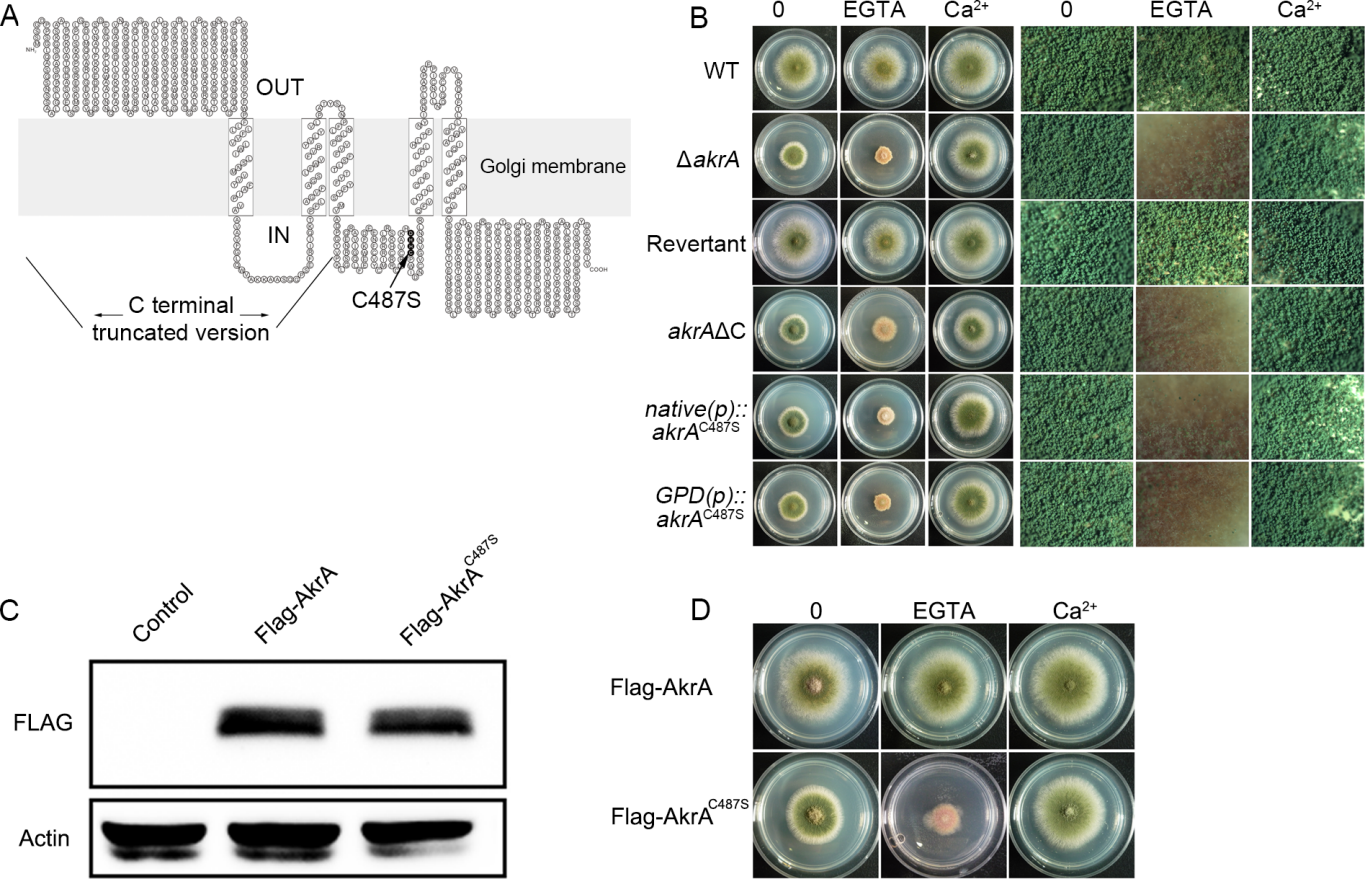
The DHHC motif is required for the function of AkrA. A. The predicted secondary structure of AkrA. It contains five predicted transmembrane domains, six ankyrin repeat sequences mapping to the NH_2_-terminal hydrophilic domain, and a DHHC-CRD sequence located between transmembrane domains 3 and 4. A hydrophobicity plot using the SOSUI program (http://harrier.nagahama-i-bio.ac.jp/sosui/) predicted a secondary amino acid structure for AkrA. The C-terminal truncated mutant and mutation site of the AkrA-C487S were labeled as indicated by the arrow. B. The colony morphology and conidiation pattern of TN02A7 (WT), Δ*akrA*, *akrA*ΔC, *native(p)*::*akrA*^C487S^ and *GPD(p)*::*akrA*^C487S^ grown on solid minimal media in the presence or absence of 1mM EGTA or 20 mM CaCl_2_, respectively, at 37°C for 2.5 days. C. Western blot analysis of total protein extracts of TN02A7 (WT), Flag-AkrA and Flag-AkrA^C487S^ strains probed with anti-Flag antibody. Anti-actin antibody against actin was used as an internal control of loading. D. Growth phenotype of indicated strains grown on solid minimal media in the presence or absence of 1mM EGTA or 20 mM CaCl_2_, respectively, at 37°C for 2.5 days.

Because the bioinformatic analysis showed that AkrA contains a conserved DHHC motif required for its palmitoylation activity [19–21], we next investigated whether the DHHC motif was required for the normal function of AkrA under low calcium conditions. We first constructed a C-terminal AkrA truncation lacking the region from the DHHC motif through to the stop codon by homologous gene replacement (Fig 3A). The colony phenotype of the truncation mutant was similar to that resulting from the complete deletion of the *akrA* gene when grown in minimal medium plus EGTA, indicating that the DHHC motif is required for AkrA function (Fig 3B). To rule out the possibility that a loss of function in the truncated mutant might result from a conformational change that prevented a true reflection of the function of the DHHC motif, we performed site-directed mutagenesis. Since Cys^487^ in the DHHC motif has previously been shown to be crucial for palmitoyl transferase activity, we therefore mutated Cys^487^ to Ser^487^ in the DHHC motif (Fig 3A) [35,36]. Consequently, we found that the C487S site-mutated DHHS fragment could not rescue the defect of the *akrA* deletion mutant under either the control of a native promoter (*native(p)*::*akrA*^C487S^) or a GPD promoter (*GPD(p)*::*akrA*^C487S^) (Fig 3B). In comparison, the wild-type *akrA* gene completely rescued the growth defects in the *akrA* deletion recipient strain. To confirm that these fusion cassettes were transcribed in the transformant, we performed quantitative real-time PCR to verify the *akrA* mRNA levels. The results showed that both the GPD and native promoters induced normal *akrA* mRNA expression, even though the mRNA expression level under the control of the GPD promoter was higher than that with the native promoter (S4D and S4E Fig), indicating that the AkrA-DHHS cassettes were fully transcribed. Next, we generated Flag-tagged AkrA and the site mutated AkrA^C487S^ strains to further confirm the expression of the AkrA protein. As shown in Fig 3C, the predicted bands on a Western blot were observed clearly, suggesting that both Flag-AkrA and Flag-AkrA^C487S^ proteins were fully expressed *in vivo*. In addition, the Flag-tagged AkrA^C487S^ strain displayed an identical phenotype to that of the Flag-untagged (*native(p)*::*akrA*^C487S^) mutant, suggesting that the Flag tag could not phenotypically change the function of the targeted protein AkrA (Fig 3B and 3D). These data suggest that the growth defect caused by *akrA* deletion was closely associated with the Cys^487^ site in the DHHC motif.

### AkrA functions independently of previously identified HACS components

Because the loss of *akrA* caused a similar defect phenotype to that of deletion mutants of the HACS components *cchA* and *midA* under the low calcium conditions, we hypothesized that AkrA forms a complex with CchA or MidA to perform its function. To assess whether AkrA physically interacts with CchA or MidA, we performed yeast two-hybrid assays. We cloned the cDNA fragments corresponding to the cytosolic C-terminus of *cchA* and the full-length cDNA of *midA*, respectively. They were then amplified and cloned into the pGADT7 vector, which contains the GAL4 DNA-AD and the LEU2 marker. In addition, a full-length cDNA of *akrA* was cloned into the pGBKT7 vector, which contains the GAL4 DNA-BD and TRP1 marker. As a result, some small colonies of pGBKT7-*akrA* with pGADT7-*cchA* were obtained, and there was no detectable growth of colonies of pGBKT7-*akrA* with pGADT7-*midA* under the high stringency screening conditions compared to the positive colonies of pGADT7-T and pGBKT7-53, which showed robust growth (S4A Fig). These data suggest that AkrA and MidA do not directly interact, and that AkrA and CchA might weakly and transiently interacted.

**Fig 4:**
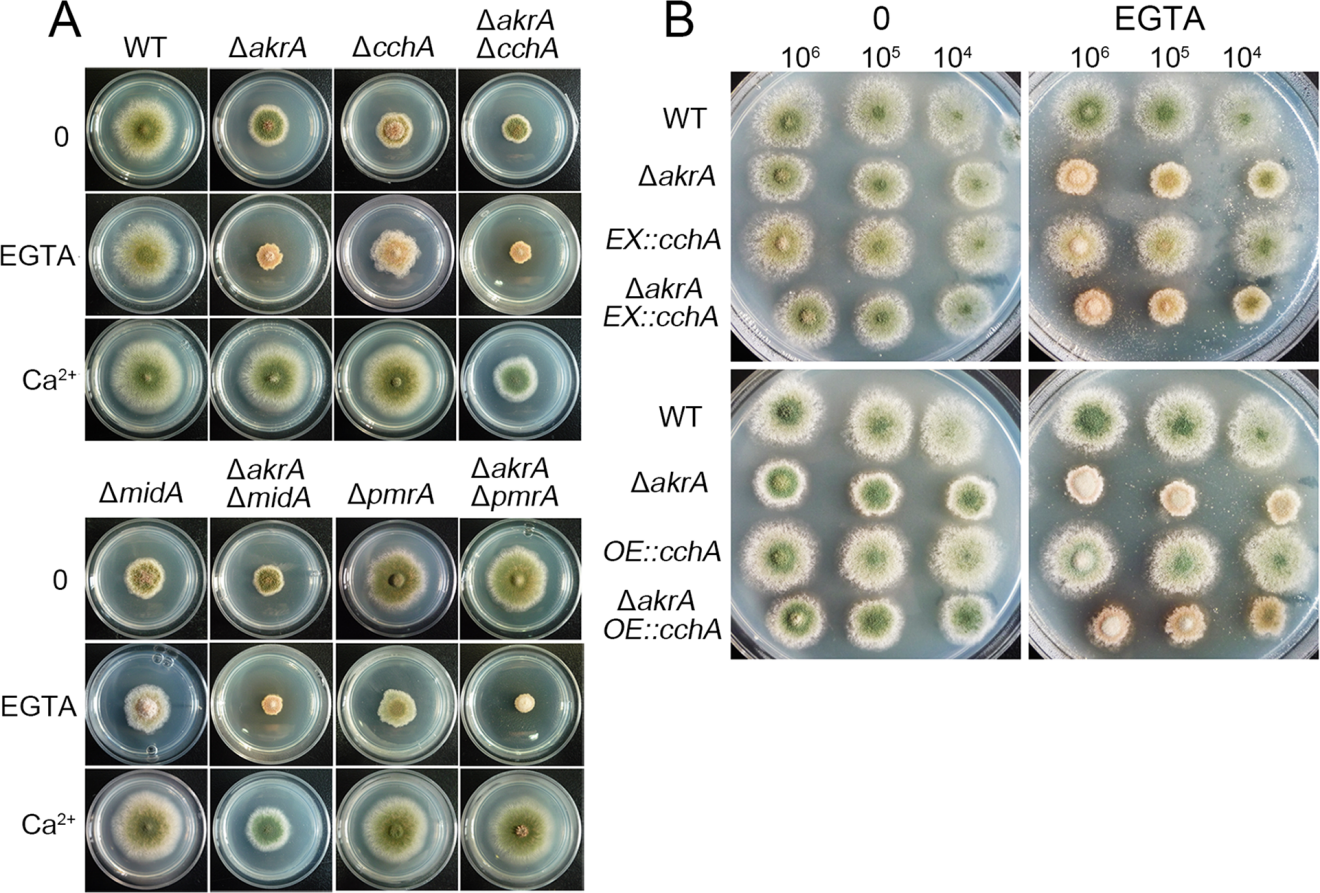
Relationship between AkrA and the CchA, MidA and PmrA. A. Colony morphology comparison for the indicated strains grown on solid minimal media in the presence or absence of 1 mM EGTA or 20 mM CaCl_2_ at 37°C for 2.5 days. B. Colony phenotypes of the indicated strains at a series of 2 μL 10-fold dilutions derived from a starting suspension of 10^6^ conidia/mL grown on solid inducing medium (upper panels) or solid overexpressing medium (lower panels) in the presence or absence of 1mM EGTA at 37°C for 2.5 days.

We next investigated the functional interaction(s) between AkrA and CchA and between AkrA and MidA by a genetic phenotypic analysis. The Δ*akrA*Δ*midA*, Δ*akrA*Δ*cchA* double mutants were generated by genetic crossing. As shown in Figs 4A and S6, phenotypic defects in colony size and conidiation were exacerbated in the double mutants compared to the parental single mutants, especially in the presence of EGTA. Notably, the growth retardation of the Δ*akrA*Δ*midA* and Δ*akrA*Δ*cchA* double mutants under low calcium conditions was reversed by the addition of 20 mM calcium to the minimal medium. These results suggest that AkrA, CchA, and MidA are all required under the calcium-limited condition, but may have some non-overlapping roles in growth. To determine whether overexpression of *cchA* could rescue the Δ*akrA* defects under the low calcium condition, we crossed Δ*akrA* (ZYA02) and *alcA(p)*::GFP*-cchA* (ZYA11) to generate the ZYA12 strain. Real-time PCR verified that the mRNA level of *cchA* in ZYA12 was approximately 15-fold higher in the overexpressing medium than in the inducing medium when cultured for 18 h (S4C Fig). However, overexpression of *cchA* did not rescue the Δ*akrA* defects under low calcium conditions (Fig 4B).

Previous studies have demonstrated that *pmr1*, which encodes a Ca^2+^/Mn^2+^ P-type ATPase and is involved in Ca^2+^ homeostasis, localizes to the Golgi in yeast [37]. In *A*. *nidulans*, Δ*pmrA* had no discernible effect on fungal physiology, but the cells were hypersensitive to low extracellular calcium [38]. To investigate the link between AkrA and PmrA, we crossed the Δ*akrA* and Δ*pmrA* mutants. Surprisingly, the double mutant had no detectable defect when grown in minimal medium compared to the Δ*akrA* strain, which had a reduced-colony size (Fig 4A). These data suggest that the *pmrA* deletion suppressed the Δ*akrA* growth defect. However, when cultured on minimal medium with 1 mM EGTA, the double mutant showed an exacerbated growth retardation phenotype compared to the parental single mutants. In addition, the phenotypic defects of Δ*akrA*Δ*pmrA* were completely suppressed by the addition of 20 mM calcium. These results suggest that AkrA and PmrA may operate together in regulating cellular calcium homeostasis in a reverse way.

### AkrA mediates the extracellular calcium-induced [Ca^2+^]_c_ transient

**Fig 5:**
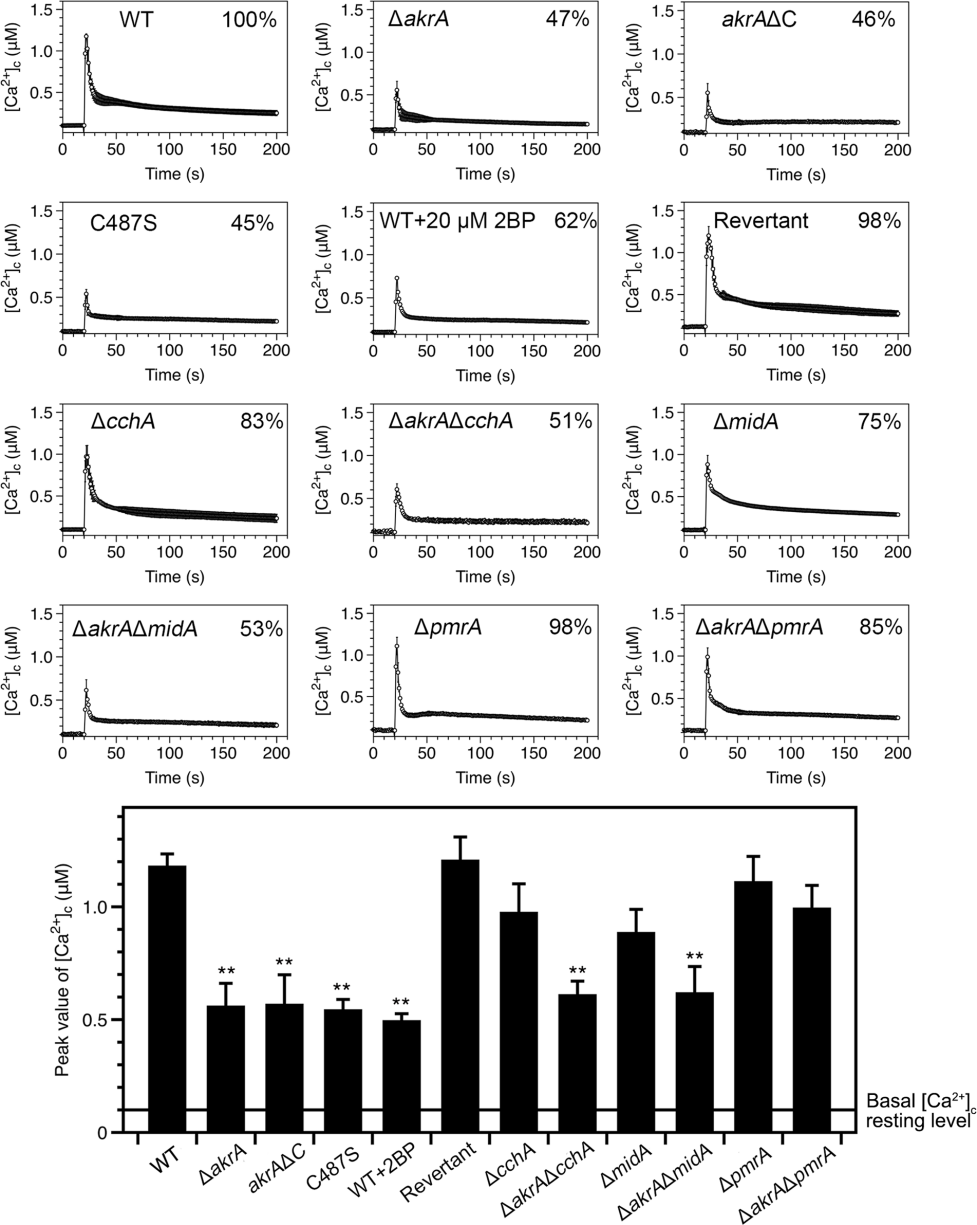
Extracellular Ca^2+^-induced [Ca^2+^]_c_ transients in *akrA* mutants. [Ca^2+^]_c_ responses in the wild type and indicated mutant strains following a stimulus of high external calcium (0.1 M CaCl_2_) with the peak [Ca^2+^]_c_ amplitudes expressed as a percentage of that of the wild-type. The bar graph shows the peak [Ca^2+^]_c_ concentrations of the indicated strains after treatment with CaCl_2_. The basal [Ca^2+^]_c_ resting level is indicated by the line (approximately 0.1 μM in these experiments), **p<0.01. Values represent averages of six wells and error bars represent SD (n = 6).

Previous studies with yeast reported that Cch1 and Mid1 mutations reduced calcium uptake and affected [Ca^2+^]_c_ accumulation under both stimulating and non-stimulating conditions [5,39–41]. We monitored the extracellular calcium-induced [Ca^2+^]_c_ changes in living cells of *A*. *nidulans* wild type and mutant strains in which we expressed codon-optimized aequorin [42–44]. When treated with 0.1 M CaCl_2_, the [Ca^2+^]_c_ concentration in wild type cells transiently increased from a resting level of approximately 0.1 μM to a peak concentration of 1.2 μM (Fig 5). In comparison, *cchA* or *midA* mutants showed a reduction of 17 ± 11% or 25 ± 12% in the [Ca^2+^]_c_ amplitudes, respectively, under the same stimulating conditions. Surprisingly, the decrease in the [Ca^2+^]_c_ amplitude in *akrA* mutants was much larger than that observed in the HACS mutants. The [Ca^2+^]_c_ amplitudes were decreased as follows: 53 ± 13% in the *akrA* deletion strain ZYA02, 54 ± 9% in the DHHC truncated mutant ZYA15, and 55 ± 8% in the site-mutated *native(p)*::*akrA*^C487S^ mutant ZYA16. These data suggest the significant reduction in calcium influx due to the loss of AkrA is mediated by the DHHC motif and, in particular, the cysteine residue within the DHHC motif.

The [Ca^2+^]_c_ amplitude in the Δ*pmrA* mutant exposed to the 0.1 M CaCl_2_ stimulus was similar to that of the parental wild-type strain, which is different from that previously reported for yeast [45–47], suggesting that other Ca^2+^-ATPases may compensate for the loss of PmrA function in response to the extracellular calcium stimulus. However, loss of *pmrA* in the *akrA* deletion background was able to recover the decreased [Ca^2+^]_c_ amplitude in the *akrA* mutant to a similar level as that in the parental wild-type strain in response this extracellular calcium stimulus, indicating that the perturbation of calcium homeostasis induced by AkrA could be rescued by loss of *pmrA*.

The protein palmitoylation inhibitor 2-bromopalmitate (2-BP) is a palmitate analog that blocks palmitate incorporation into proteins [48,49]. To determine whether inhibition of palmitoyl transferase activity influences calcium influx into the cytoplasm, we measured the [Ca^2+^]_c_ amplitude of the wild type pre-incubated in 2-BP (20 μM) for 2 h. Following this drug treatment, the amplitude of the [Ca^2+^]_c_ increase following stimulation with 0.1 M CaCl_2_ was significantly reduced by approximately 40% of the untreated cells in response to stimulation with 0.1 M CaCl_2_ (Fig 5). These data suggest that the inhibition of palmitoyl transferase activity can significantly block calcium influx.

### Loss of AkrA abolishes [Ca^2+^]_c_ responses to ER or plasma membrane stress

**Fig 6:**
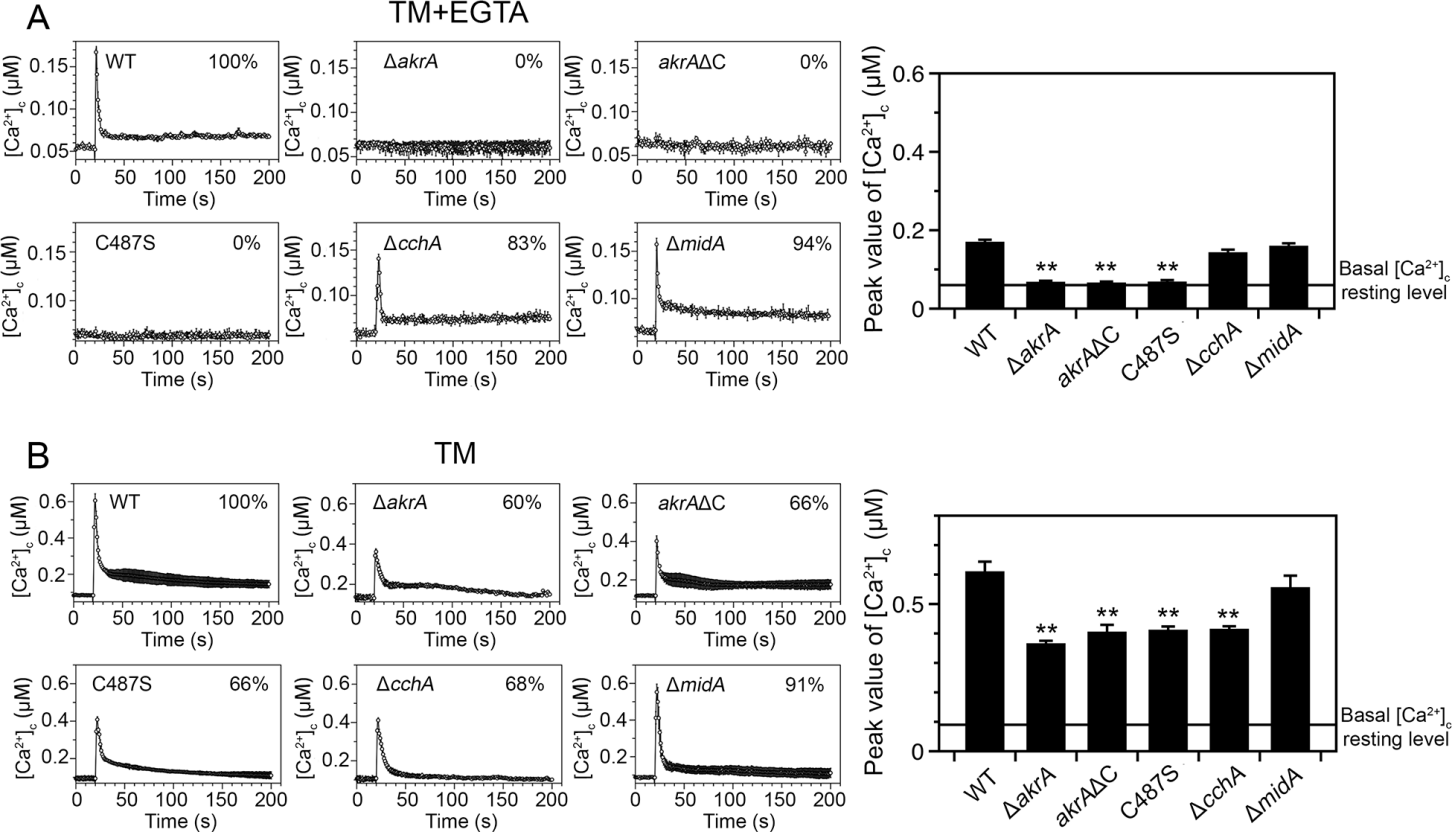
AkrA regulates the [Ca^2+^]_c_ transient induced by ER stress following tunicamycin treatment. A. [Ca^2+^]_c_ responses in the wild type and indicated mutant strains to 5 μg/mL tunicamycin pretreated for 10 min with the calcium chelator EGTA (1 mM). The peak [Ca^2+^]_c_ amplitudes are expressed as a percentage of that of the wild-type. The bar graph shows the peak [Ca^2+^]_c_ concentrations of the indicated strains after treatment with EGTA and Tunicamycin (TM) (right panel). The basal [Ca^2+^]_c_ resting level is indicated by the line (approximately 0.08 μM in these experiments), **p<0.01. In each experiment, values represent averages of six wells and error bars represent SD (n = 6). B. [Ca^2+^]_c_ responses in the wild type and indicated mutant strains to 5 μg/mL TM. The bar graph shows the peak [Ca^2+^]_c_ concentrations of the indicated strains after treatment with TM (right panel). The basal [Ca^2+^]_c_ resting level is indicated by the line (approximately 0.1 μM in these experiments), **p<0.01.

Activation of Ca^2+^ channels, calmodulin, calcineurin and other factors is necessary for the long-term survival of cells undergoing ER stress, and during this process the HACS components, CchA and MidA, are required for Ca^2+^ influx from the extracellular environment [41,50,51]. To verify whether AkrA is involved in the calcium influx response during ER stress, we measured the influence of the ER-stress agents, tunicamycin (TM) and dithiothreitol (DTT) on [Ca^2+^]_c_. When the parental wild-type strain was treated with 5 μg/mL tunicamycin, we observed an immediate transient increase in [Ca^2+^]_c_ with an amplitude of 0.60 ± 0.03 μM (Fig 6B). In comparison, the [Ca^2+^]_c_ amplitude in the Δ*cchA* mutant (but not the Δ*midA* mutant) in response to tunicamycin was decreased by approximately 32 ± 6%, suggesting that the loss of CchA but not MidA mediates the ER stress-induced calcium influx in *A*. *nidulans*. Furthermore, in response to tunicamycin treatment the [Ca^2+^]_c_ amplitude decreased by 40 ± 5%, 34 ± 8% and 34 ± 6% in the Δ*akrA*, *akrA*ΔC, *native(p)*::*akrA*^C487S^ mutants, respectively. We next examined the [Ca^2+^]_c_ response after addition of DTT, another agent causing ER-stress. 10 mM DTT induced a rapid increase in [Ca^2+^]_c_ which peaked at approximately 0.40 μM in the wild-type and Δ*midA* strains, but the [Ca^2+^]_c_ amplitudes decreased by approximately 40% in the Δ*akrA* (36 ± 10%), *akrA*ΔC (37 ± 7%), and *native(p)*::*akrA*^C487S^ (36 ± 8%) mutants, and by 15 ± 9% in the Δ*cchA* mutant (S7 Fig). These data suggest that CchA, but not MidA, influences the ER stress-induced calcium influx in *A*. *nidulans*, which is different from that previously reported in yeast [41,51]. Furthermore, loss of AkrA, or mutations in its DHHC significantly decreased the ER stress-induced calcium influx. We further tested whether the amplitude of the [Ca^2+^]_c_ increase in response to tunicamycin was dependent on the extracellular calcium concentration. We found that there was no significant change when mycelia were cultured in media with or without 5 mM calcium (S8A Fig). In contrast, exposure of cells to 1 mM EGTA prior to tunicamycin treatment completely abolished the increase in [Ca^2+^]_c_ in the Δ*akrA*, *akrA*ΔC and *native(p)*::*akrA*^C487S^ mutants, but not in the parental wild-type, Δ*cchA* or Δ*midA* strains (Fig 6A). Similar data was obtained when we used the more selective, calcium chelator BAPTA (S9 Fig). These data suggest that intracellular calcium stores contribute to the transient increase in [Ca^2+^]_c_ induced by agents causing ER stress.

**Fig 7:**
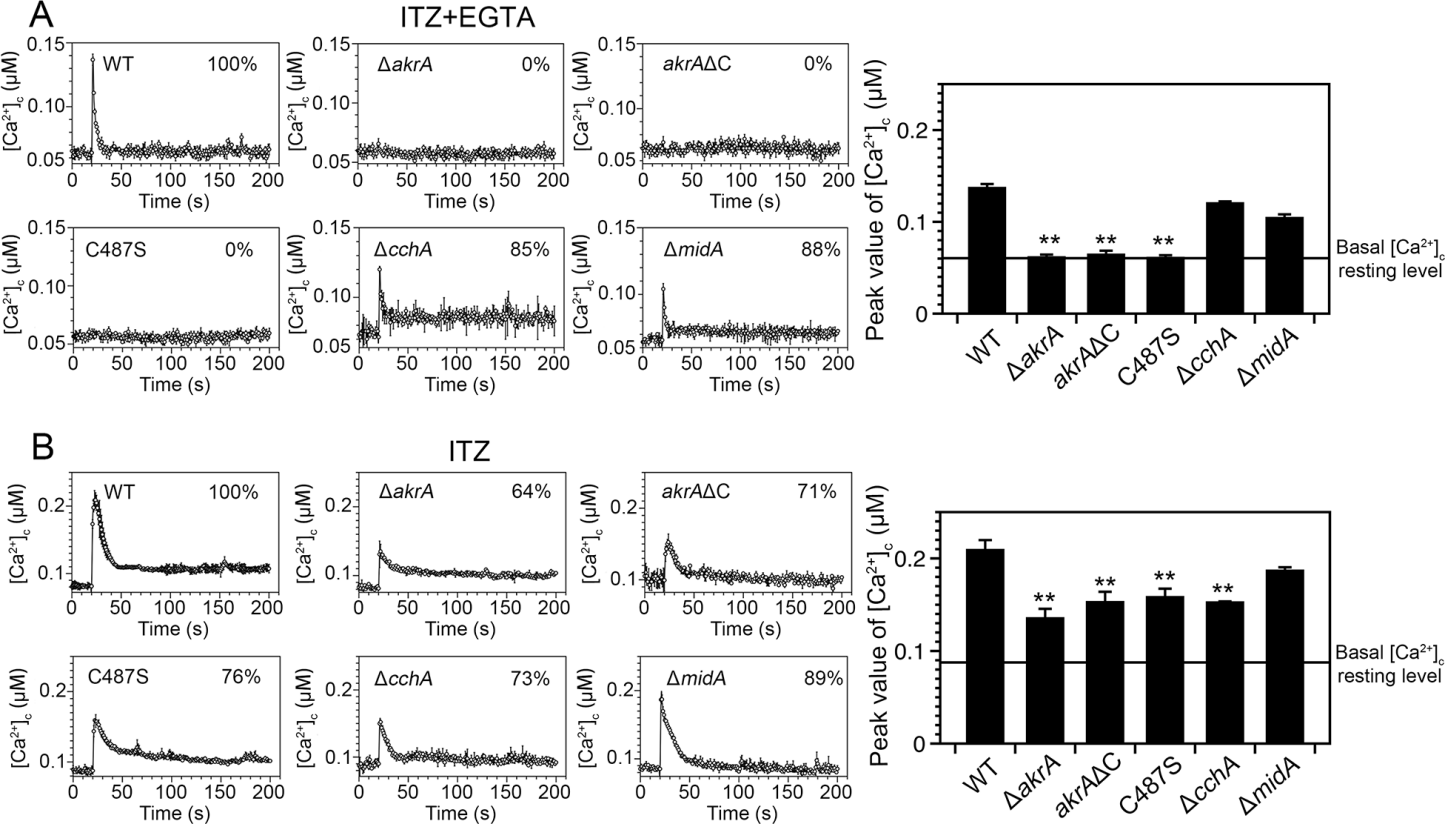
*akrA* regulates the [Ca^2+^]_c_ transient induced by plasma membrane stress following antifungal azole treatment. A. [Ca^2+^]_c_ responses in the indicated strains to ITZ (1 μg/mL) pretreated for 10 min with the calcium chelator EGTA (1 mM). The peak [Ca^2+^]_c_ amplitudes are expressed as a percentage of that of the wild-type. The bar graph shows the peak [Ca^2+^]_c_ concentrations of the indicated strains after treatment with EGTA and ITZ (right panel), **p<0.01. The basal [Ca^2+^]_c_ resting level is indicated by the line (approximately 0.06 μM in these experiments). In each experiment, values represent averages of six wells and error bars represent SD (n = 6). B. [Ca^2+^]_c_ responses to ITZ (1 μg/mL) in the indicated strains. The bar graph shows the peak [Ca^2+^]_c_ concentrations of the indicated strains after treatment with ITZ (right panel), **p<0.01. The basal [Ca^2+^]_c_ resting level is indicated by the line (approximately 0.09 μM in these experiments).

Because azole antifungal drugs induce plasma membrane stress [13,14,52], we next compared the differences in the [Ca^2+^]_c_ transient between wild-type and relevant mutant strains after treatment with the azole antifungal agent itraconazole (ITZ), which is currently used as a primary antifungal drug in the clinic. In all the tested mutants and the wild-type strain, the [Ca^2+^]_c_ resting levels were similar at approximately 0.05 μM. After addition of 1 μg/mL ITZ to the medium, all strains responded with a transient increase in [Ca^2+^]_c_ (Fig 7B). However, all the *akrA* defective mutants exhibited significantly lower increases in [Ca^2+^]_c_ compared to their parental wild-type strain: the amplitudes of the [Ca^2+^]_c_ transients were reduced by 36 ± 11% in the Δ*akrA*, 29 ± 10% in the *akrA*ΔC, 24 ± 8% in the *native(p)*::*akrA*^C487S^ and 27 ± 8% in the Δ*cchA* mutants, respectively, compared to that of the parental wild-type strain. In marked contrast to these mutants, the Δ*midA* mutant exhibited a similar [Ca^2+^]_c_ amplitude in response to ITZ as observed in the wild-type strain. In addition, the amplitude of the ITZ-induced [Ca^2+^]_c_ elevation increased when mycelia were cultured in media containing 5 mM CaCl_2_ (S8B Fig). We next examined whether the [Ca^2+^]_c_ transient induced in response to ITZ was dependent on external calcium or internal calcium stores. We exposed hyphal cells to media supplemented with EGTA (1 mM) prior to ITZ treatment, and found that [Ca^2+^]_c_ transients were dramatically abolished in all the Δ*akrA* mutants, whereas the [Ca^2+^]_c_ transients in the wild type, and the Δ*cchA* and Δ*midA* mutants, were still observed (Fig 7A). Similar data were obtained when we used the calcium chelator BAPTA (S9 Fig). These data indicate that the loss of AkrA or disruption of its DHHC motif in the absence of extracellular calcium completely block calcium influx after treatment with chemicals that induce ER or plasma membrane stress from both extracellular and intracellular sources. Furthermore, both extracellular calcium and intracellular calcium stores play roles in generating these [Ca^2+^]_c_ transients induced by these stress treatments.

### The cysteine residue of the DHHC motif is required for AkrA palmitoylation

**Fig 8:**
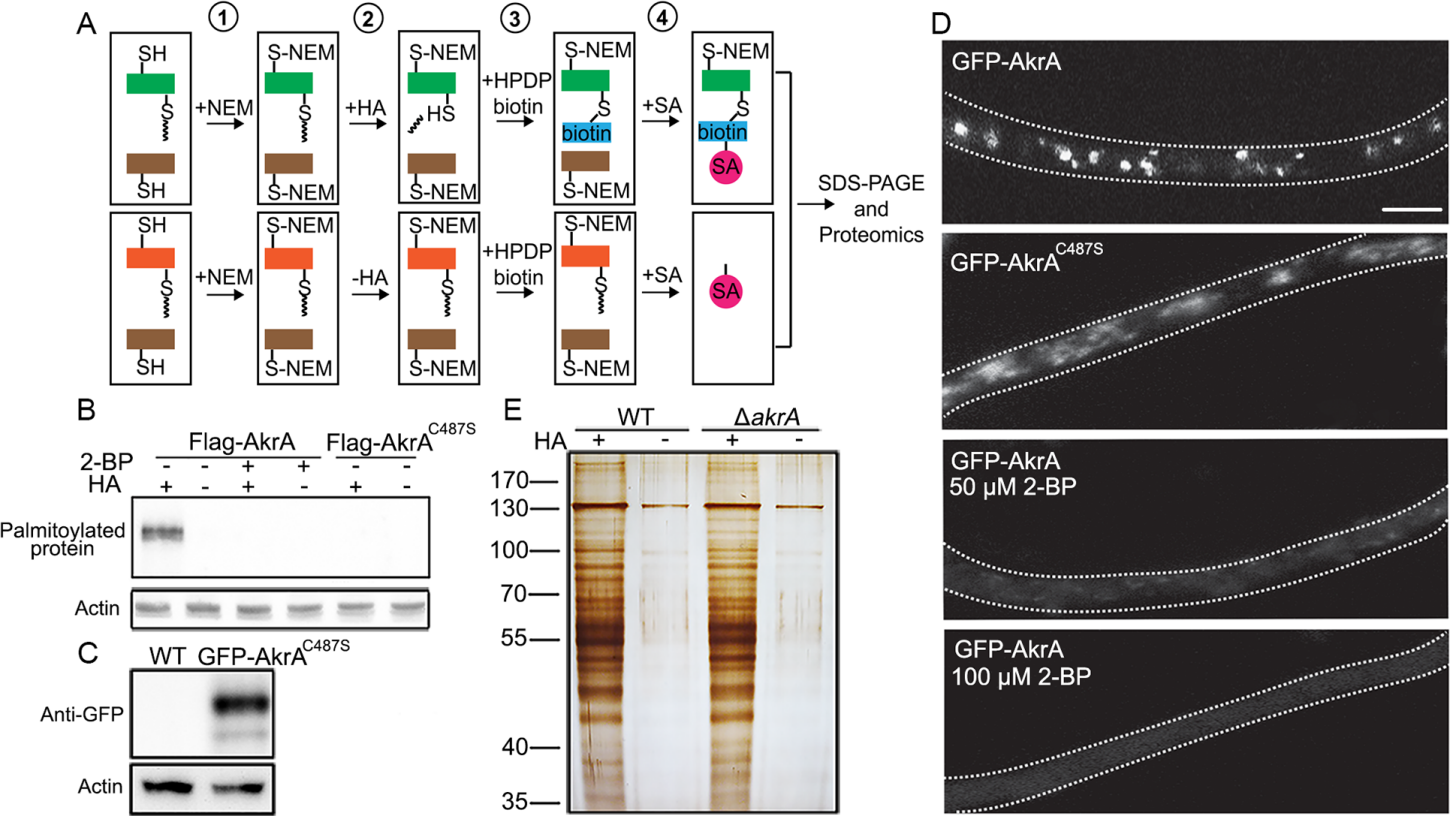
The cysteine residue in the DHHC motif, is correspondingly required for AkrA palmitoylation. A. A schematic diagram of the acyl-biotin exchange (ABE) assay. ① Blocking the free sulfhydryls with N-ethylmaleimide (NEM); ② Cleavaging the thioester bonds with or without hydroxylamine (HA); ③ Biotinylating the palmitoylated proteins with HPDP-biotin; Lastly, ④ Enriching the biotinylated proteins bound to streptavidin agarose (SA). B. Flag-AkrA and Flag-AkrA^C487S^ were detected by Western blotting with anti-Flag antibodies using the ABE assay, treated or not with 100 μM 2-bromopalmitate (2-BP). Hydroxylamine (HA) was used to specifically cleave S-acyl groups revealing sulfhydryl groups, which were subsequently labeled with biotin. Samples were then bound to streptavidin beads. For the negative control HA was substituted by Tris. Anti-actin antibody was used as an internal control of loading. A band was detected in the +HA treated sample, indicating that it was bound to an acyl group via a thioester linkage confirming that it is auto-acylated. However, no signal was detected for Flag-AkrA^C487S^ and 2-BP treatment samples and therefore they are not auto-acylated. C. Western blot analysis indicated a fusion protein of GFP-AkrA^C487S^ was detected with a predicted size of approximately 100 kDa by using an anti-GFP antibody. D. GFP-AkrA and GFP-AkrA^C487S^ localization was assessed after culturing for 18 h in liquid induced medium supplemented with or without the indicated concentration of 2-BP. Localization within the Golgi was less distinct as punctate structures in the GFP-AkrA^C487S^ strain compared with that in the wild-type and its localization within the Golgi was completely abolished after 2-BP treatment. Bar, 2 μm. E. Total proteins from wild type and Δ*akrA* strains subjected to the ABE assay with (HA+) or without (HA-) hydroxylamine treatment. The samples were then electrophoresed by SDS-PAGE and detected by silver nitrate staining.

Our evidence above indicates that the cysteine residue in the DHHC motif of AkrA is involved in regulating the calcium response to high extracellular calcium-, ER- and plasma membrane-stress. To test whether the cysteine residue of DHHC is required for AkrA palmitoylation, we set up an acyl-biotin exchange (ABE) chemistry assay to detect palmitoylation in potential target proteins based on selective thioester hydrolysis by hydroxylamine (HA) (Fig 8A). Compared to the control, the treatment of hydroxylamine combined with N-ethylmaleimide (NEM) (which blocks free sulhydryls), efficiently enriches palmitoylated proteins. Subsequent treatment with HA cleaves the thioester bond between palmitate and cysteine residues, exposing bound thiols, which are then covalently linked to HPDP-biotin. The controls were protein samples not treated with HA. Lastly, the biotinylated proteins were bound to streptavidin agarose, washed with buffer, and eluted by cleavage of the cysteine-biotin disulfide linkage following by SDS-PAGE. Several previous reports have suggested that the process of palmitoylation involves in a two-step mechanism in which palmitoyl transferase is auto-acylated by itself to create an intermediate followed by the transfer of the palmitoyl moiety to its substrate [53,54]. Therefore, to investigate whether the cysteine residue in the DHHC motif is responsible for AkrA auto-acylation, we used the ABE assay to detect whether AkrA palmitoylates itself [20]. As shown in Fig 8B, when HA was present, Flag-AkrA can be clearly detected with an anti-Flag antibody. However, a site-directed mutation of the cysteine residue in the DHHC motif and the parental wild-type strain pre-cultured with 2-bromopalmitate (2-BP) completely abolished palmitoylation of AkrA, which resulted in no signal being detected in the enriched pamitoylated proteins. These results indicate that AkrA is able to be auto-acylated and the cysteine residue in the DHHC motif is required for this process. In addition, we found that treatment with 2-BP (24 h, 50 and 100 μM) virtually abolished the Golgi localization of GFP-labelled AkrA (Fig 8D) and resulted in a similar defective growth defect phenotype to the Δ*akrA* mutant on minimal medium (S10 Fig).

We constructed another *alcA(p)*::GFP*-akrA*^C487S^ mutant and confirmed by Western blotting (Fig 8C) to further check whether site directed mutagenesis of the Cys^487^ in the DHHC motif disrupted the normal localization of AkrA in the Golgi. The GFP-AkrA^C487S^ was less distinctly localized in the punctate Golgi structures characteristic of wild-type GFP-AkrA and some appeared to be localized in the cytoplasm (Fig 8D). These data collectively suggest that the cysteine residue in the DHHC motif of AkrA and the palmitoylation activity are closely associated with AkrA auto-acylation, which is required for normal AkrA localization and palmitoylation.

To further explore palmitoylated protein substrates specifically mediated by AkrA, total proteins of the wild-type and Δ*akrA* strains were treated and analyzed using the ABE chemistry assay combined with liquid chromatograpy-mass spectrometry (LC-MS) for comparative proteomics (Fig 8E). Using this approach, 334 proteins were identified as potential AkrA substrates in the parental wild-type strain because they were completely absent in the Δ*akrA* strain. As shown in Table 1, AkrA belonged to one of the AkrA-mediated pamitoylated substrates suggesting it is able to auto-acylate itself. Among the palmitoylated protein candidates identified, Yck2, Lcb1, Ras2, Cdc48 and Pab1 have been previously identified as palmitoylated proteins in *S*. *cerevisiae* but only Yck2 has been characterized as an Akr1 substrate [20,55–57]. These data indicated that the ABE chemistry assay combined with LC-MS was a valid approach to identify proteins palmitoylated by AkrA and it also indicated that *A*. *nidulans* may palmitoylate some of the substrates previously reported in *S*. *cerevisiae*. In our study we notably identified the following protein substrates palmitoylated by AkrA: a vacuolar Ca^2+^-ATPase Pmc1 homolog (AN5088.4); a P-type ATPase Spf1 homolog (AN3146.4) involved in calcium homeostasis [58]; a putative V-type H^+^-ATPase Vma5 homolog (AN1195.4) that has been linked to Ca^2+^-ATPase function [59], and three uncharacterized proteins (AN8774.4, AN3420.4 and AN2427.4), the transcripts of which have previously been shown to be induced by extracellular calcium stress in a CrzA-dependent manner [53]. These results provide strong evidence that the AkrA protein regulates [Ca^2+^]_c_ homeostasis in *A*. *nidulans* by palmitoylating these protein candidates. Other candidate substrates of AkrA that we identified included the P450 enzymes, Cyp51A (Erg11A), Cyp51B (Erg11B) and Erg5 homologs, which are all involved in ergosterol biosynthesis and azole resistance. Thus AkrA may influence the azole resistance by these biosynthetic enzymes.

**Table 1:** Selected *A*. *nidulans* palmitoylated proteins.

Accession Number	Systematic Name	Description	*S*.*cerevisiae* gene
**Calcium-signalling/homeostasis related proteins**			
**CBF76107.1**	AN5088	P-type Ca^2+^-ATPase	*pmc1*
**XP_658799.1**	AN1195	Vacuolar P-type H^+^-ATPase	*vma5*
**XP_660750.1**	AN3146	Putative P-type Ca^2+-^ATPase involved in ER function and homeostasis	*spf1*
**CBF78046.1**	AN8774	Transcript induced in response to CaCl_2_ in a CrzA-dependent manner	*upc2*
**CBF82745.1**	AN3420	Transcript induced in response to CaCl_2_ in a CrzA-dependent manner	*lys14*
**XP_660031.1**	AN2427	Transcript induced in response to CaCl_2_ in a CrzA-dependent manner	*Unnamed*
**Ergosterol biosynthetic proteins**			
**CBF85786.1**	AN1901	Putative sterol 14 alpha-demethylase	*cyp51A (erg11A)*
**CBF74274.1**	AN8283	Putative sterol 14-demethylase	*cyp51B (erg11B)*
**CBF73688.1**	AN8004	Putative cytochrome P450	*erg5*
**CBF74719.1**	AN4094	Putative C-14 sterol reductase	*erg24*
**CBF84799.1**	AN1409	Putative acetyl-CoA C-acetyltransferase	*erg10*
**Other proteins**			
**CBF81263.1**	AN5757	Putative casein kinase-type protein kinase	*yck2*
**XP_663428.1**	AN5824	Ortholog(s) have palmitoyltransferase activity and role in protein palmitoylation	*akr1*
**XP_664146.1**	AN6542	Gamma-actin	*act1*
**AAK40365.1**	AN3728	Serine palmitoyltransferase, target of an antifungal drug, myriocin	*lcb1*
**CBF74428.1**	AN4234	Putative phosphoacetylglucosamine mutase with a predicted role in chitin biosynthesis	*pcm1*
**CBF78760.1**	AN7254	Protein with a conserved CDC48, cell division protein N-terminal domain and two ATPase domains of the AAA-superfamily	*cdc48*
**CBF70756.1**	AN5832	Putative Ras GTPase	*ras2*
**Q5B630.2**	AN4000	Protein with similarity to poly(A)-binding proteins; overexpression results in increased brlA expression and asexual development;	*pab1*

## Discussion

Palmitoylation is a reversible post-translational modification that is involved in regulating the trafficking and the functional modulation of membrane proteins. Many proteins that rely on palmitoylation are key players in cellular signaling, membrane trafficking and synaptic transmission [19–21]. Yeast Akr1p was the first characterized palmitoyl transferase (PAT) [36,60]. AkrA, a human AkrA homolog HIP14, is involved in palmitoylation and plays an important role in the trafficking of multiple neuronal proteins associated with Huntington’s disease [61]. Calcium serves a multitude of signaling and structural functions in all eukaryotes. Recent studies in mammalian systems have shown that the skeletal muscle ryanodine receptor/Ca^2+^-release channel RyR1 is subject to S-palmitoylation modification in ''hot spot'' regions containing sites of mutations implicated in malignant hyperthermia and central core disease [62]. However, studies on the relationship between calcium signaling components and palmitoylation are very scarce. In this study, we identified that homologs of the yeast palmitoyl transferase in *A*. *nidulans* (AkrA) and *A*. *fumigatus* (*Af*AkrA) are required for hyphal growth and sporulation under low external calcium conditions. High extracellular calcium-, ER- and plasma membrane-stress conditions all elicited transient increases in [Ca^2+^]_c_. These [Ca^2+^]_c_ responses were all mediated by AkrA and involved the cysteine residue in its DHHC motif, which was shown to be required for AkrA palmitoylation. Candidate protein substrates that the AkrA protein is involved in palmitoylating were found to include many key components involved in membrane trafficking and cellular signaling processes including known palmitoylated Ras-like proteins (Table 1). Among them were: a vacuolar Ca^2+^ ATPase Pmc1 homolog [63]; a putative P-type ATPase Spf1 homolog, which is involved in ER function and calcium homeostasis in budding yeast and *Candida albicans* [58,64,65]; a Vma5 homolog that has been linked with Pmr1 Ca^2+^-ATPase function [59], and three calcium signaling- related proteins (encoded by AN8774.4, AN3420.4 and AN2724.4), the transcripts of which have been previously shown to be induced in response to high extracellular calcium stress which is dependent on the transcription factor CrzA [53]. Key P450 enzymes in the ergosterol biosynthesis pathway were also identified as AkrA palmitoylated proteins. Thus, our findings suggest that mutation of the DHHC motif in AkrA results in the disruption of [Ca^2+^]_c_ homeostasis that is mainly due to the absence of the post-translational, palmitoylated-modification of key proteins involved in calcium signaling/homeostasis. PmcA and SpfA are homologs of two Ca^2+^ ATPases which response for sequestrating calcium into intercellular compartments in *S*. *cerevisiae* [63,64].

### AkrA mediates the [Ca^2+^]_c_ transient in response to high extracellular calcium stress independently of the CchA/MidA complex

**Fig 9:**
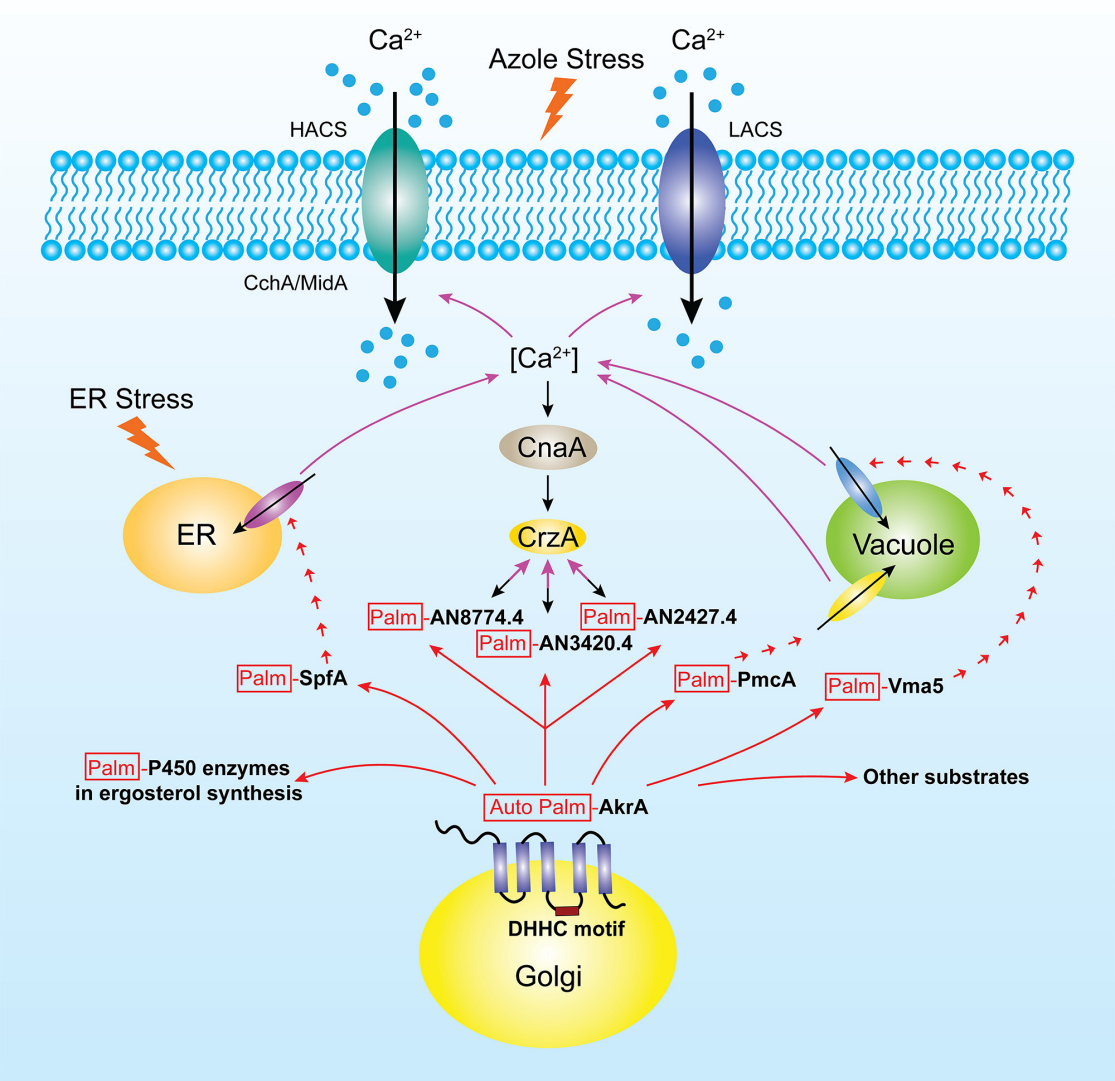
A working model of how AkrA function regulates [Ca^2+^]_c_ homeostasis in *A*. *nidulans*. AkrA protein mediates [Ca^2+^]_c_ homeostasis by palmitoylating protein candidates labeled by Palm: a putative P-type ATPase Spf1 homolog, a calcium ion transport Vma5 homolog and three uncharacterized proteins, the transcripts of which are induced in response to extracellular calcium stress in a CrzA-dependent manner in *A*. *nidulans*.

Deletion of the *akrA* gene exhibited marked growth and conidiation defects under low calcium conditions, which is similar to the defects caused by mutations in the CchA/MidA HACS [28–30]. In addition, the *akrA* deletion conferred increased sensitivity to Li^+^, Na^+^, K^+^, Mg^2+^, but slightly increased resistance to the cell wall disrupting agents compared to the parental wild-type strain (S5 Fig). Moreover, the Δ*akrA*Δ*cchA* and Δ*akrA*Δ*midA* double mutants exacerbated the Δ*akrA* defects under calcium-limited conditions, suggesting that AkrA may have independent functions to those of the CchA-MidA complex. AkrA localized to trans Golgi structures (Fig 2C), while the CchA-MidA complex probably localizes to the plasma membrane as reported for yeast [40,66,67]. In addition, results from the Y2H assays (S4A Fig) suggested that there were no direct, or only very weak, interactions between AkrA and CchA and between AkrA and MidA. Nevertheless, the [Ca^2+^]_c_ transient in the Δ*akrA* mutant had a much lower amplitude (approximately 53 ± 13% lower) than the wild-type control following treatment with a high extracellular calcium stress stimulus, suggesting that the loss of AkrA reduced calcium influx into the cytoplasm. In contrast, loss of CchA and MidA caused a 25% decrease in the [Ca^2+^]_c_ amplitude in response to this treatment with high external calcium, consistent with the results from previous studies on yeast cells lacking either Cch1 or Mid1, which exhibited a low calcium uptake [5,39–41]. The *akrA* deletion also had a bigger impact on inhibiting calcium influx in response to ER stress than observed in the Δ*cchA* and Δ*midA* mutants. Overall our data suggests that AkrA regulates calcium uptake from the external medium as well and its release from intracellular Ca^2+^ stores through a pathway that is independent of the previously identified CchA/MidA HACS as shown in Fig 9. PmrA is an *A*. *nidulans* homolog of yeast Pmr1, which is a P-type Golgi Ca^2+^/Mn^2+^ ATPase responsible for Ca^2+^ transport into the Golgi and widely accepted as responsible for Ca^2+^ efflux from the cytoplasm into the Golgi to regulate calcium signaling and homeostasis and prevent calcium toxicity. Loss of Pmr1 function in budding yeast is believed to inhibit the return of [Ca^2+^]_c_ to its resting level following stimulus-induced [Ca^2+^]_c_ increases [37,45–47]. In contrast, our data showed that the *pmrA* deletion in *A*. *nidulans* exhibited no significant change in the calcium signature following a high extracellular calcium stress stimulus compared with the wild-type strain, suggesting that other paralogs of *pmrA* (*e*.*g*. other Ca^2+^-ATPases) may compensate or play more important roles in returning the elevated [Ca^2+^]_c_ back to its resting level. Surprisingly, loss of *pmrA* alleviated the decreased response of the Δ*akrA* mutants to the external calcium stimulus, resulting in the amplitude of the [Ca^2+^]_c_ increase of the double mutant Δ*pmrA*Δ*akrA* being almost back to the normal level of the wild type. Thus deletion of PmrA reverses the effects of the AkrA deletion in regulating calcium influx following extracellular calcium stress. The lower amplitude of the [Ca^2+^]_c_ increase of the Δ*akrA* mutant in response to the high extracellular calcium stimulus indicate that AkrA and its pamitoylated targets play a role in mediating the calcium influx into the cytoplasm and then PmrA may store cytoplasmic calcium into Golgi. When both PmrA and AkrA were absent, the increase in [Ca^2+^]_c_ following extracellular calcium stimulation was back to almost the normal level in the wild-type (Fig 5). This suggests that the [Ca^2+^]_c_ increase in the Δ*pmrA*Δ*akrA* double mutant following treatment with high extracellular calcium is compensated by some other unknown component(s) of the calcium signaling/homeostatic machinery. Furthermore, our data (Fig 4A) showed that loss of *pmrA* suppressed the colony growth defect of Δ*akrA* mutants, providing further evidence to support interactive regulatory roles of PmrA and AkrA in *A*.*nidulans*.

Previous studies have verified that exposure of fungi to ER or plasma membrane stress stimulates store-operated calcium influx through the HACS to promote fungal cell survival [13,14,41,50–52]. Consistent with previous studies, in *A*. *nidulans* we observed a transient increase in [Ca^2+^]_c_ after treatment with the ER-stress agents tunicamycin (TM) or dithiothreitol (DTT). The Δ*cchA* mutant exhibited reduced [Ca^2+^]_c_ amplitudes by 32 ± 6% and 15 ± 9% upon treatment with TM or DTT, respectively (Figs 6 and S7). In contrast, we did not detect a change in the [Ca^2+^]_c_ response to the ER stress agents in the Δ*midA* mutant compared to its parental wild-type strain. This suggests that as a complex of CchA and MidA, CchA may have a more predominant role than MidA during the ER stress response. Moreover, the Δ*akrA* mutant displayed a decreased response to ER and plasma membrane stress inducing drugs, as the [Ca^2+^]_c_ amplitude of Δ*akrA* mutants decreased by approximately 36–40% of the wild-type strain following treatment with these drugs (Figs 6 and S7). These data suggest that, in addition to HACS components, AkrA is also involved in ER and plasma membrane stress-induced calcium influx. Moreover, these responses were completely abolished in the Δ*akrA* mutant but not in the wild-type strain in the presence of EGTA or BAPTA that chelate external calcium. These results indicate that both extracellular calcium and calcium stores contribute to the transient [Ca^2+^]_c_ changes following ER or plasma membrane stress. Because calcium release from intracellular stores in response to these types of stress was abolished in the *akrA* mutants (Figs 6, 7 and S9), our results are consistent with AkrA regulating calcium influx across the plasma membrane, which in turn activates the release of calcium from intracellular pools.

Altogether, our results provide the first report that AkrA is a putative palmitoyl transferase in *A*. *nidulans*, and it mediates calcium influx in a DHHC-dependent mechanism to perform an essential function in calcium homeostasis/signaling for survival under high extracellular calcium-, ER- or azole antifungal-stress conditions.

Calcium signaling regulators have been previously identified as antifungal target candidates, including FK506, which targets calcineurin [8]. However, most of the fungal homologs of known calcium signaling components in mammalian cells are of proteins also required for mammalian cell growth and metabolism [68]. Thus, potential antifungals against these components may cause side effects in mammalian hosts. The use of drugs that target regulators of posttranslational modification of calcium signaling that show significant differences to their mammalian homologs (*e*.*g*. AkrA only exhibits 24.8% identity to the human AkrA homolog HIP14), may circumvent this problem. The potential for developing novel antifungal drugs of this type has been greatly facilitated by our study that has shown a critical link between palmitoylation and calcium signaling.

### The [Ca^2+^]_c_ response is closely associated with the cysteine residue in the DHHC motif which is correspondingly required for AkrA palmitoylation

Previous studies have shown that all AkrA homologs across different species require the DHHC motif to be active and function normally as palmitoyl transferases [69–71]. Three approaches were initially employed to determine AkrA function: deletion of the DHHC motif; site-directed mutagenesis of the cysteine residue in the DHHC motif; and use of a specific palmitoyl transferase analogue inhibitor (2-bromopalmitate), to determine AkrA function [48,49]. Our data from these experiments suggested that the DHHC motif and its cysteine residue are required for the function of AkrA, especially when extracellular calcium is limited. To further test whether the cysteine residue in the DHHC motif, is correspondingly required for AkrA palmitoylation, we used the acyl-biotin exchange (ABE) chemistry assay to detect palmitoylation based on selective thioester hydrolysis by hydroxylamine. Compared to the treatment without hydroxylamine, the newly exposed cysteine residues are disulfide-bonded to a biotin analogue, affinity purified and digested into peptides, leaving the labeled peptides on the affinity beads so that palmitoylated proteins have been enriched. As the ABE chemistry detects palmitoylation through identification of all the thioester linkages. A subsequent Western experiment was used to further confirm palmitoylated proteins by specific antibodies. Consequently, among these enriched palmitoylated proteins, Flag-AkrA was clearly detected with an anti-Flag antibody. Site-directed mutation of the cysteine residue in the DHHC or treatment of the parental wild-type strain with the palmitoyl transferase analogue inhibitor 2-BP completely abolished palmitoylation of AkrA (Fig 8B). Previous studies have demonstrated that although the exact mechanism of S-acylation is not known, palmitoylation of the purified DHHC-CRD palmitoylated proteins zDHHC2, zDHHC3 and yeast Erf2, involves a two-step mechanism, in which the zDHHCs form an acyl-enzyme intermediate (auto-acylation), with the acyl group later transferred to the target protein [53,54]. Our results indicated that AkrA auto-acylated itself before palmitoylating its target proteins. In mammalian cells, any protein that contains a surface-exposed and freely accessible cysteine that has transient access to Golgi membranes is susceptible to palmitoylation. Our data suggests AkrA both auto-acylated itself and palmitoylates target proteins in association with Golgi membranes. Moreover, we found that site directed mutagenesis of the Cys^487^ in the DHHC motif significantly affect normal localization of AkrA in the Golgi. When we treated cells with a specific palmitoyl transferase analogue inhibitor 2-BP, AkrA localization within the Golgi localization was completely lost (Fig 8D), suggesting that the 2-BP treatment not only prevented AkrA auto-acyltation but also prevented the normal subcellular localization of AkrA. The reason for the different localization pattern, if any, caused by the site directed mutagenesis and the treatment of 2-BP as shown in Fig 8D is likely to be due to a side effect of the 2-BP reagent.

In conclusion, our results provide the first report that AkrA is a palmitoyl transferase in *A*. *nidulans*, and that it mediates calcium influx in a DHHC-dependent mechanism to perform an essential role in calcium homeostasis to survive high extracellular calcium-, ER- and plasma membrane-stress conditions. A working model of AkrA function in regulating [Ca^2+^]_c_ homeostasis in *A*. *nidulans* is presented in Fig 9. Our findings provide new insights into the link between palmitoylation and calcium signaling that may be of relevance for understanding the mechanistic basis of human PAT-related diseases. Regulators of posttranslational modification in fungi may provide promising targets for new therapies against life threatening fungal diseases.

## Materials and Methods

### Strains, media, and cultural conditions

All fungal strains used in this study are listed in S1 Table. Minimal media (MM), and MMPDR (minimal media + glucose + pyrodoxine + riboflavin), MMPDR+UU (minimal media + glucose + pyrodoxine + riboflavin+ uridine + uracil), MMPGR (minimal media + glycerol + pyrodoxine + riboflavin) have been described previously [29,72]. MMPGRT was MMPGR with 100 mM threonine. Fungal strains were grown on minimal media at 37°C, harvested using sterile H_2_O and stored for the long-term in 50% glycerol at −80°C. Expression of tagged genes under the control of the *alcA* promoter was regulated by different carbon sources: non-induced by glucose, induced by glycerol and overexpressed by glycerol with threonine. Growth conditions, crosses and induction conditions for *alcA(p)*-driven expression were as previously described [73].

### Construct design and tagging of AkrA with GFP

In order to generate constructs for *akrA* null mutant (Δ*akrA*), the fusion PCR method was used as previously described [74]. Primers used to design constructs are listed in S2 Table. The *A*. *fumigatus pyrG* gene in plasmid pXDRFP4 was used as a selectable nutritional marker for fungal transformation. The transformation was performed as previously described [75].

For creating an Δ*akrA* construct, a 5′ flank and a 3′ flank DNA fragments were amplified using the primers akrA-P1 and akrA-P3, akrA-P4 and akrA-P6, respectively, using genomic DNA (gDNA) of the *A*. *nidulans* wild-type strain TN02A7 as the template for PCR. As a selectable marker, a 2.8 kb DNA fragment of *A*. *fumigatus pyrG* was amplified from the plasmid pXDRFP4 using the primers pyrG-5’ and pyrG-3’. The three PCR products were combined and used as a template to generate a 4.8 kb DNA fragment using the primers akrA-P2 and akrA-P5. The final PCR product was transformed into a wild-type strain. A similar strategy was used to construct *akrA*-truncated mutants.

To design the revertant strain construct, a 3.7 kb DNA fragment, which included a 1.2 kb promoter region, a 2.4 kb coding sequence, and a 3′ flank was amplified using the primers primer A and primer D from *A*. *nidulans* gDNA. As a selectable marker, a 1.7 kb *pyroA* fragment was amplified from the plasmid pQa-pyroA using the primers pyro-5’ and pyro-3’. The two PCR products were co-transformed into the Δ*akrA* strain to produce the revertant strain.

To generate the *alcA(p)*::GFP*-akrA* vector, a 1 kb *akrA* fragment was amplified from the gDNA in the wild-type strain TN02A7 with primers akrA-5’ and akrA-3’ (S2 Table) and then ligated into the plasmid vector pLB01 yielding plasmid pLB-*alcA(p)*::GFP*-akrA* which contains GFP-N under the control of the *alcA* promoter with the *N*. *crassa pyr4* as a marker.

For site-directed mutation, a 3.7 kb *akrA* DNA fragment with a site directed mutation in which cysteine^487^ was replaced by serine and a selective marker *pyroA* were co-transformed into the Δ*akrA* strain to obtain *native(p)*::*akrA*^C487S^ strain. The fragment containing the site mutation was amplified with two steps. First, fragment AB and fragment CD were amplified from *A*. *nidulans* gDNA with primers A and B, primers C and D, respectively, and complementary regions contained the desired mutation (cysteine^487^ to serine^487^). Second, using fragment AB and fragment CD as a template, the final 3.7 kb fragment was generated through fusion PCR using primer A and primer D.

The *GPD(p)*::*akrA*^C487S^ and *alcA(p)*::GFP*-akrA*^C487S^ strains were constructed using a similar strategy. In brief, the GPD promoter was amplified with the GPD-5’ and GPD-3’, and 2.4 kb *akrA* DNA fragment including a 2.4 kb coding sequence, and a 0.5 kb 3’ flanking was amplified with akrA-GPD-5’ and primer D. These two fragments were combined using GPD-5’ and primer D, Lastly, the aboved fusion PCR products and the selective marker *pyroA* were co-transformed into the Δ*akrA* strain to obtain the *GPD(p)*::*akrA*^C487S^ strian. For the *alcA(p)*::GFP*-akrA*^C487S^ construction, a 5′ flank and a 3′ flank DNA fragments were amplified from genomic DNA of *alc-akrA* mutant using the primers alc-up and primer B, primer C and new primer D, respectively. Then the two PCR products were combined and used as a template to generate a 3.9 kb DNA fragment using the primers alc-up and new primer D, and then this fragment was ligated into a plasmid vector yielding the pEA-C487S. The *pyroA* fragment was amplified from the pQa-pyroA using the primers pyro-cre-5’ and pyro-cre-3’, then recombined into the plasmid pEA-C487S. Finally the plasmid was transformed into the Δ*akrA* strain to obtain the *alcA(p)*::*akrA*^C487S^ strian.

All N-terminal Flag constructs were designed and fabricated using restriction-free cloning protocols outlined at http://www.rf-cloning.com using PrimerSTAR MAX DNA polymerase (TAKARA, R045A) [76]. Then, N-Flag tagged cassettes and selective marker *pyroA* were co-transformed into the Δ*akrA* strain.

For the mutants expressing the codon-optimized aequorin, the plasmid pAEQS1-15 containing codon-optimized aequorin and selective markers *pyroA* or *riboB* genes were co-transformed into the indicated mutants. Transformants were screened for aequorin expression using methods described previously [77] and high aequorin expressing strains were selected after homokaryon purification involving repeated plating of single conidia.

### Plate assays

For each experiment, at least three replicate plates were used to test phenotypes for each strain. To assess the influence by the extracellular calcium to the colony phenotype, minimal medium was supplemented with 20 mM CaCl_2_ or 1 mM EGTA, respectively. The influence of osmotic stress or ionic stress was tested by adding 600 mM NaCl, 600 mM KCl, 10 mM MnCl_2_, 400 mM MgCl_2_, 400 mM CaCl_2_ or 300 mM LiCl into minimal medium, respectively. For the cell wall integrity test, the reagent of 60 μg/mL Calcofluor White or 100 μg/mL Congo Red was added to the minimal medium, respectively. 2 μL of conidia from the stock (1×10^6^ conidia/mL) for indicated strains were spotted onto relevant media and cultured for 2.5 days, at 37°C, and then the colonies were observed and imaged.

### Fluorescence microscopy observations

For microscopic observations, conidia were inoculated onto pre-cleaned glass coverslips overlaid with liquid media. To observe co-localization of GFP-AkrA and mRFP-PH^OSBP^, strain ZYA13 (S1 Table) was cultured at 37°C for 10 h in non-inducing medium (non-inducing conditions for the *alcA(p)* driving expression of AkrA) and shifted for 6 h to the inducing medium (in which the *alcA* promoter was induced) before microscopic observation [34]. Differential interference contrast (DIC) and fluorescence images of the cells were captured with a Zeiss Axio imager A1 microscope (Zeiss, Jena, Germany) equipped with a Sensicam QE cooled digital camera system (Cooke Corporation, Germany). The images were processed with MetaMorph/MetaFluor software (Universal Imaging, West Chester, PA) and assembled in Adobe Photoshop (Adobe, San Jose, CA).

### Germination assay

Germination was assessed in liquid non-inducing medium at 37°C with a total number of 10^6^ conidia/mL for each strain in their stationary phase [78]. The percentage rate of germination was measured at 4, 5, 6, 7 and 8 h by microscopic examination. Spores were considered as germinated ones when length of the germ tube was almost equal to the conidium in diameter. For each strain, three replicates of 100 spores were quantified at each time point to determine the germination rate.

### Yeast two-hybrid assay

*Saccharomyces cerevisiae* strain AH109 (Clontech, Palo Alto, CA) was used as the host for the two-hybrid interaction experiments. The analysis was performed using the Matchmaker Library Construction & Screening system (BD Clontech). For strain generation, a cDNA fragment corresponding to the cytosol C-terminus of *cchA* and the full-length cDNA of *midA* were amplified and cloned into the pGADT7 vector, which contains the GAL4 DNA-AD and the LEU2 marker (BD Clontech). Full-length cDNA of *akrA* were used for the pGBKT7 vector (Clotech, Palo Alto, CA).

### [Ca^2+^]_c_ measurement

The strains expressing the codon-optimized aequorin gene were grown on minimal media for 2.5 days to achieve maximal conidiation. 10^6^ spores with liquid media were distributed to each well of a 96-well microtiter plate (Thermo Fischer, United Kingdom). Six wells were used in parallel for each treatment. The plates were incubated at 37°C for 18 h. The medium was then removed and the cells in each well were washed twice with PGM (20 mM PIPES pH 6.7, 50 mM glucose, 1 mM MgCl_2_). Aequorin was reconstituted by incubating mycelia in 100 μL PGM containing 2.5 μM coelenterazine *f* (Sigma-Aldrich) for 4 h, at 4°C in the dark. After aequorin consititution, mycelia were washed twice with 1 mL PGM and allowed to recover to room temperature for 1 h [79,80]. To chelate extracellular Ca^2+^, 1 mM EGTA or 8 mM BAPTA was added to each well 10 min prior to stimulus injection.

At the end of each experiment, the active aequorin was completely discharged by permeabilizing the cells with 20% (vol/vol) ethanol in the presence of an excess of calcium (3 M CaCl_2_) to determine the total aequorin luminescence of each culture. Luminescence was measured with an LB 96P Microlumat Luminometer (Berthold Technologies, Germany), which was controlled by a dedicated computer running the Microsoft Windows-based Berthold WinGlow software. Conversion of luminescence (relative light units [RLU]) into [Ca^2+^]_c_ was done using Excel 2007 software (Microsoft). The relative light units (RLU) values were converted into [Ca^2+^]_c_ concentrations by using the following empirically derived calibration formula: pCa = 0.332588 (-log k) + 5.5593, where k is luminescence (in RLU) s^-1^/total luminescence (in RLU) [77]. Error bars represent the standard error of the mean of six independent experiments, and percentages in the figures represent peak of [Ca^2+^]_c_ compared to that of the wild-type (100%).

### Acyl-biotin exchange (ABE) assay and mass spectrometry

ABE was performed as described previously with some modifications [81]. Briefly, the strain mycelium was ground to a fine powder in liquid nitrogen and resuspended in 5 mL lysis buffer. Samples were incubated for 1 h at 4°C followed by centrifugation at 4°C, 13,000 g to remove insoluble material. 5 mg of protein was incubated overnight with 50 mM N-ethylmaleimide (NEM) at 4°C to reduce proteolysis while allowing free sulhydryls to be blocked. Proteins were precipitated at room temperature using methanol/chloroform. The pellet was resuspended in 200 μL resuspension buffer and the solution divided into two equal aliquots. One aliquot was combined with 800 μL of 1 M fresh hydroxylamine (HA), 1 mM EDTA, protease inhibitors and 100 μL 4 mM biotin-HPDP (Thermo Scientific). As a control the remaining aliquot was treated identically but hydroxylamine (HA) was replaced with 50 mM Tris pH 7.4. Proteins were precipitated and resuspended in 100 μL of resuspension buffer. 900 μL PBS containing 0.2% Triton X-100 was added to each sample, aliquots were removed as a loading control, and the remaining reactions were incubated with 30 μL of streptavidin-agarose beads (Thermo scientific). The streptavidin beads were washed four times with 1 mL PBS containing 0.5 M NaCl and 0.1% SDS. Proteins were eluted by heating at 95°C in 40 μL 2× SDS sample buffer containing 1% 2-mercaptoethanol v/v. Samples were analyzed by silver staining or Western blotting as described below. In some cases, cells were treated with 50 or 100 μM of the palmitoylation inhibitor 2-bromopalmitate (2-BP) before the ABE assay.

For mass spectrometry (MS), total protein (100 μg) extracted from each sample was chemically reduced for 1 h at 60°C by adding DTT to 10 mM and carboxyamidomethylated in 55 mM iodoacetamide for 45 min at room temperature in the dark. Then trypsin gold (Promega, Madison, WI, USA) was added to give a final substrate/enzyme ratio of 30:1 (w/w). The trypsin digest was incubated at 37°C for 16 h. After digestion, the peptide mixture was acidified by 10 μL of formic acid for further MS analysis. After protein digestion, each peptide sample was desalted using a Strata X column (Phenomenex), vacuum-dried and then resuspended in a 200 μL volume of buffer A (2% ACN, 0.1% FA). After centrifugation at 20000 g for 10 min, the supernatant was recovered to obtain a peptide solution with a final concentration of approximately 0.5 μg/μL. 10 μL supernatant was loaded on a LC-20AD nano-HPLC (Shimadzu, Kyoto, Japan) by the autosampler onto a 2 cm C18 trap column. The peptides were then eluted onto a 10 cm analytical C18 column (inner diameter 75 μm) packed in-house. The samples were loaded at 8 μL/min for 4 min, then the 35 min gradient was run at 300 nL/min starting from 2 to 35% buffer B (95% ACN, 0.1% FA), followed by a 5 min linear gradient to 60%, then followed by a 2 min linear gradient to 80%, and maintenance at 80% buffer B for 4 min, and finally returned to 5% in 1 min.

Data acquisition was performed with a TripleTOF 5600 System (AB SCIEX, Concord, ON) fitted with a Nanospray III source (AB SCIEX, Concord, ON) and a pulled quartz tip as the emitter (New Objectives, Woburn, MA). Data was acquired using an ion spray voltage of 2.5 kV, curtain gas of 30 psi, nebulizer gas of 15 psi, and an interface heater temperature of 150. The MS was operated with a RP of greater than or equal to 30,000 FWHM for TOF MS scans. Raw data files acquired from the Orbitrap were converted into MGF files using Proteome Discoverer 1.2 (PD 1.2, Thermo), [5,600 msconverter] and the MGF file were searched. Protein identification was performed by using Mascot search engine (Matrix Science, London, UK; version 2.3.02) against a database containing 13,597 sequences.

### Western blotting analysis

To extract proteins from *A*. *nidulans* mycelia, conidia from *alcA(p)*::GFP*-akrA* and the wild-type strains were inoculated in the liquid inducing medium, then shaken at 220 rpm on a rotary shaker at 37°C for 24 h. The mycelium was ground in liquid nitrogen with a mortar and pestle and suspended in ice-cold extraction buffer (50 mM HEPES pH 7.4, 137 mM KCl, 10% glycerol containing, 1 mM EDTA, 1 μg/mL pepstatin A, 1 μg/mL leupeptin, 1 mM PMSF). Equal amounts of protein (40 μg) per lane were subjected to 10% SDS–PAGE, transferred to PVDF membrane (Immobilon-P, Millipore) in 384 mM glycine, 50 mM Tris (pH 8.4), 20% methanol at 250 mA for 1.5 h, and the membrane was then blocked with PBS, 5% milk, 0.1% Tween 20. Next, the membrane was then probed sequentially with 1:3000 dilutions of the primary antibodies anti-GFP or anti-FLAG or anti-actin and goat anti-rabbit IgG-horseradish peroxidase diluted in PBS, 5% milk, 0.1% Tween 20. Blots were developed using the Clarity ECL Western blotting detection reagents (Bio-Rad), and images were acquired with the Tanon 4200 Chemiluminescent Imaging System (Tanon).

### RNA preparation and quantitative RT-PCR

The mycelia were cultured for 18 h in liquid media and were then ground to a fine powder in liquid nitrogen. Total RNA was isolated using Trizol (Invitrogen, 15596–025) following the manufacturer’s instructions. 100 mg of mycelia per sample was used as the starting material for the determination of total RNA. The reverse transcription polymerase chain reaction (RT-PCR) was carried out using HiScript Q RT SuperMix (Vazyme, R123-01), and then cDNA was used for the real-time analysis. For real-time reverse transcription quantitative PCR (RT-qPCR), independent assays were performed using SYBR Premix Ex Taq (TaKaRa, DRR041A) with three biological replicates, and expression levels normalized to the mRNA level of *actin*. The 2^-ΔCT^ method was used to determine the change in expression.

## Supporting Information

S1 Fig Constructions of *akrA* deletion and conditional strains. A. Diagram illustrating the targeted gene homologous replacement for the *akrA* gene (left panel). Diagnostic PCR confirmed the homologous integration at the original *akrA* locus in the Δ*akrA* strain (right panel). B. Diagram showing the strategy for *alcA(p)*::GFP-*akrA* strain (left panel). Diagnostic PCR confirmed the homologous integration at the original *akrA* locus at the *alcA(p)*::GFP-*akrA* strain (right panel).
(TIF)

S2 Fig Deletion of the *akrA* homolog gene-*AfakrA* in *A*. *fumigatus* caused hypersensitivity to low-calcium conditions. A. The colony morphology of Af1160 (WT) and Δ*AfakrA* strains grown on minimal medium at 37°C for 2.5 days in the presence or absence of 5 mM EGTA or 20 mM CaCl_2_. B. Quantitative data for the number of conidia and the colony diameters in different treatments related to panel A. All the indicated strains were grown on minimal medium in the presence of 5 mM EGTA or 20 mM CaCl_2_. Error bars represent standard deviation of three replicates, **p<0.01.
(TIF)

S3 Fig Quantitative data of the growth patterns for *akrA* deletion mutant. A. Comparison of mycelial extension rates of TN02A7 (WT), Δ*akrA* and revertant strains. Over 7 days, the colony diameter of each culture from spot-inoculated strains (each inoculum containing ~ 200 spores) was measured daily. B. Comparison of fungal biomass in liquid minimal medium after strains had been cultured for 24 h at 37°C, 220 rpm. Mycelia were dried and their dry weight was measured, **p<0.01. C. Germination rates in TN02A7 (WT), Δ*akrA* and revertant strains. Conidia were incubated in stationary liquid minimal media at the times indicated. 100 conidia for each strain were assessed for germination. These experiments were performed in triplicate, and the results are displayed as mean values with standard errors. D. Comparison of conidial germination of the TN02A7 (WT) and Δ*akrA* mutant imaged by differential interference contrast (DIC) microscopy after incubation for 7 h.
(TIF)

S4 Fig Yeast two-hybrid assay and mRNA expression analysis of the indicated strains. A. Physical interaction assay among AkrA, CchA and MidA revealed by yeast two-hybrid system. A cDNA fragment corresponding to the cytosol C-terminus of CchA and the full-length cDNA of MidA were placed in frame with the DNA activation domain of GAL4 in the pGADT7 while the full-length cDNA of AkrA was cloned to pGBKT7 vector. Protein–protein interactions were detected by growth in high-stringency media for selection (SD/-Ade/-His/-Leu/-Trp). pGADT7-T and pGBKT7-p53 were used together as a control for the interaction. B. Expression analysis by quantitative PCR of *akrA* using the *alcA* conditional promoter in liquid media (MMPDR, MMPGR and MMPGRT) providing non-inducing, inducing and overexpressing conditions, respectively. All mRNA levels were normalized to the mRNA level of *actin* (*actA*). The error bars indicate the standard deviation for three independent replicates, **p<0.01. C. Expression analysis of *cchA* using the *alcA* conditional promoter in liquid induced medium, overexpressed medium by quantitative PCR. All mRNA levels were normalized to an mRNA level of *actin* (*actA*). The error bars indicate the standard deviation for three independent replicates, **p<0.01. D, E. Expression of *akrA* using the *akrA* native or GPD promoter was examined using semi-quantitative PCR (D) and quantitative real-time PCR (E) from *native(p)*::*akrA*^*C487S*^ and *GDP(p)*::*akrA*^*C487S*^ strains. All mRNA levels were normalized to the mRNA level of *actin* (*actA*). The error bars indicate the standard deviation for three independent replicates, **p<0.01.
(TIF)

S5 Fig Loss of AkrA displayed more sensitive phenotypes to other cations and slight resistance to cell wall disturbing agents. Phenotypic comparison of TN02A7 (WT), Δ*akrA* and revertant strains in minimal medium in the presence of the indicated cations and cell wall disrupting agents Congo Red and Calcofluor White. A series of 2 μL 10-fold dilutions derived from a starting suspension of 10^7^ conidia/mL as indicated were spotted onto solid minimal medium supplemented with 0.3 M Li^+^, 0.6 M K^+^, 0.6 M Na^+^, 0.4 M Mg^2+^, 60 μg/mL Calcofluor White and 100 μg/mL Congo Red, respectively, and incubated at 37°C for 2.5 days.
(TIF)

S6 Fig Quantitative data of conidial number and colony diameters for indicated strains. All the indicated strains were grown on the minimal medium in the presence of 1 mM EGTA or 20 mM CaCl_2_. Error bars represent standard deviation of three replicates, **p<0.01.
(TIF)

S7 Fig Dithiothreitol-induced changes in [Ca^2+^]_c_ transients in *akrA* mutants. [Ca^2+^]_c_ responses in the indicated strains to dithiothreitol (DTT) (10 mM). In each experiment, values represent averages of six wells and error bars represent SD (n = 6).
(TIF)

S8 Fig Changes in [Ca^2+^]_c_ transients induced by tunicamycin and itraconazole. [Ca^2+^]_c_ responses in the indicated strains to (A) tunicamycin (5 μg/mL) and (B) ITZ (1 μg/mL) supplemented with 5 mM CaCl_2_. Values represent averages of six well and error bars represent SD (n = 6).
(TIF)

S9 Fig [Ca^2+^]_c_ transient changes induced by tunicamycin and itraconazole are abolished by the addition of BAPTA. The effect of pretreatment with the calcium chelator BAPTA (8 mM) on the [Ca^2+^]_c_ transient induced by tunicamycin (5 μg/mL) and itraconazole (1 μg/mL) is shown. In each experiment, values represent averages of six well and error bars represent SD (n = 6).
(TIF)

S10 Fig The parental wild-type strain precultured with 2-bromopalmitate shows a similar growth defect phenotype to Δ*akrA* on minimal medium. The colony morphology of TN02A7 (WT) and Δ*akrA* strains in a series of 2 μL 10-fold dilutions derived from a starting suspension of 10^6^ conidia/mL grown on minimal medium at 37°C for 2.5 days in the presence or absence of 100 μM 2-bromopalmitate (2-BP).
(TIF)

S1 Table Strains used in this study. (DOCX)

S2 Table Primers used in this study. (DOCX)
