# Supplementary figures and images for: Palmitoylation of the Cysteine Residue in the DHHC Motif of a Palmitoyl Transferase Mediates Ca2+ Homeostasis in Aspergillus
Source: PLoS Genet. 2016 Apr 8;12(4):e1005977. doi: 10.1371/journal.pgen.1005977 (PMC4825924; doi:10.1371/journal.pgen.1005977)

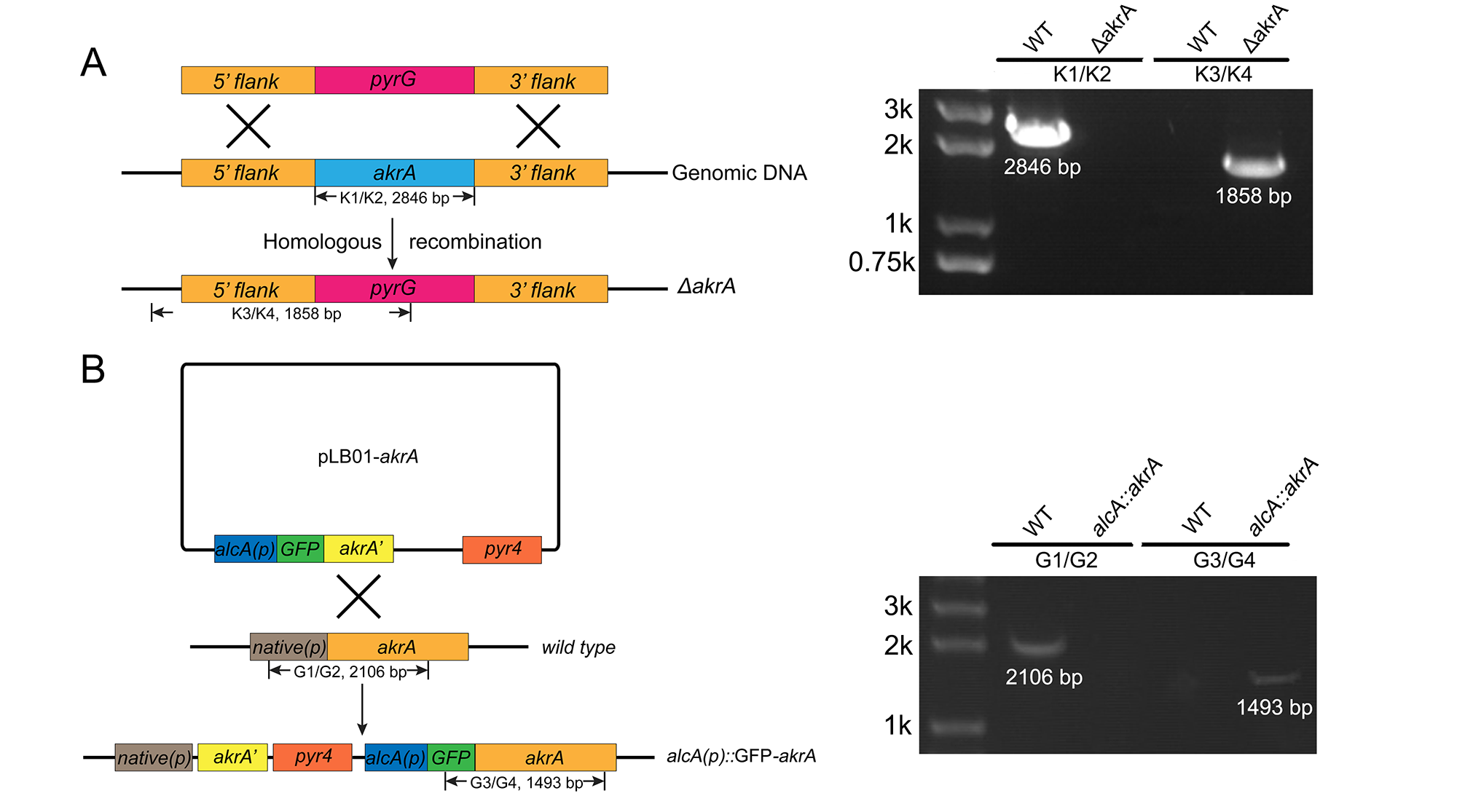

Supplement: S1 Fig — A. Diagram illustrating the targeted gene homologous replacement for the akrA gene (left panel). Diagnostic PCR confirmed the homologous integration at the original akrA locus in the ΔakrA strain (right panel). B. Diagram showing the strategy for alcA(p)::GFP-akrA strain (left panel). Diagnostic PCR confirmed the homologous integration at the original akrA locus at the alcA(p)::GFP-akrA strain (right panel). (TIF) [file pgen.1005977.s001.tif]

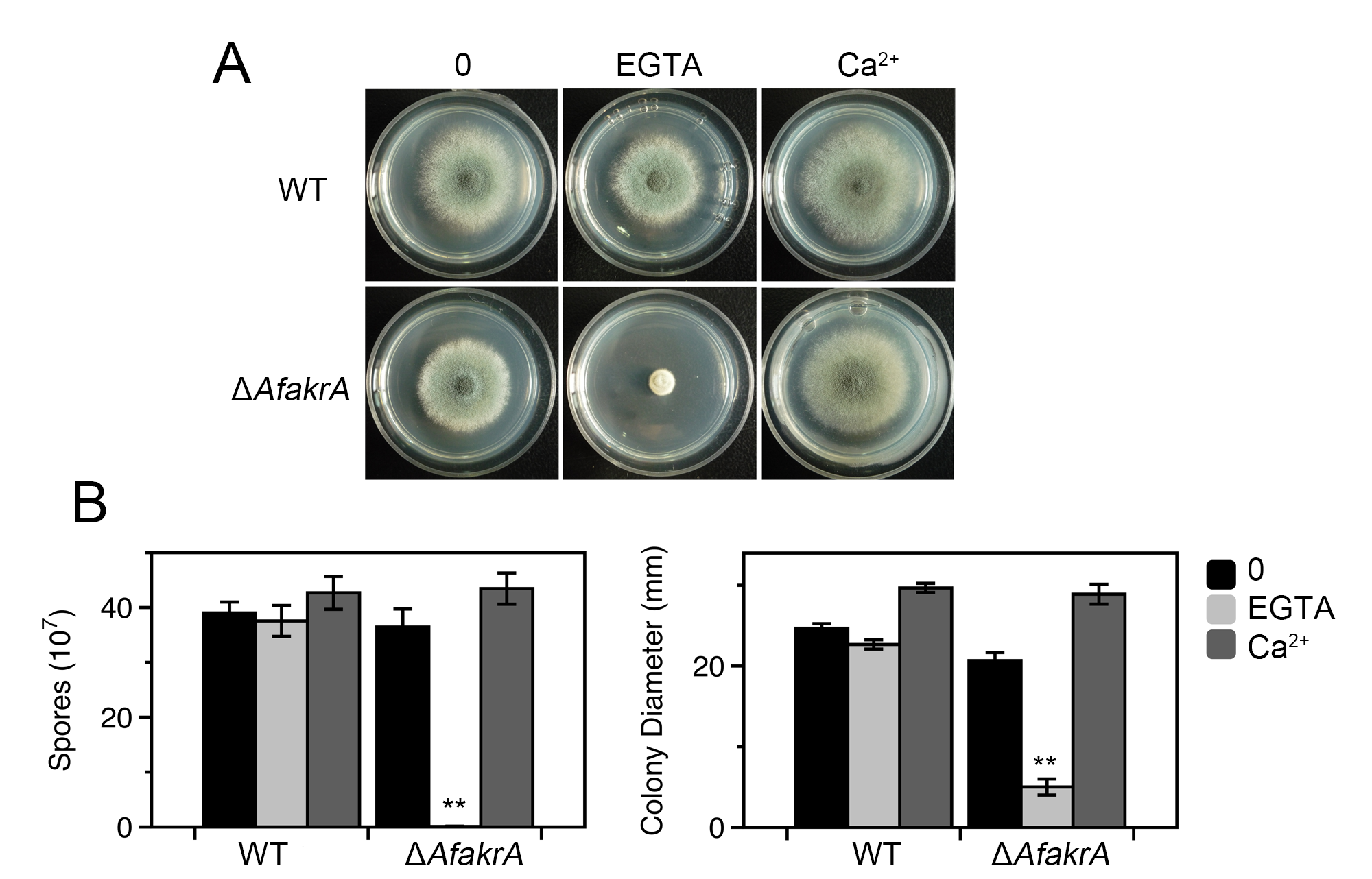

Supplement: S2 Fig — A. The colony morphology of Af1160 (WT) and ΔAfakrA strains grown on minimal medium at 37°C for 2.5 days in the presence or absence of 5 mM EGTA or 20 mM CaCl2. B. Quantitative data for the number of conidia and the colony diameters in different treatments related to panel A. All the indicated strains were grown on minimal medium in the presence of 5 mM EGTA or 20 mM CaCl2. Error bars represent standard deviation of three replicates, **p<0.01. (TIF) [file pgen.1005977.s002.tif]

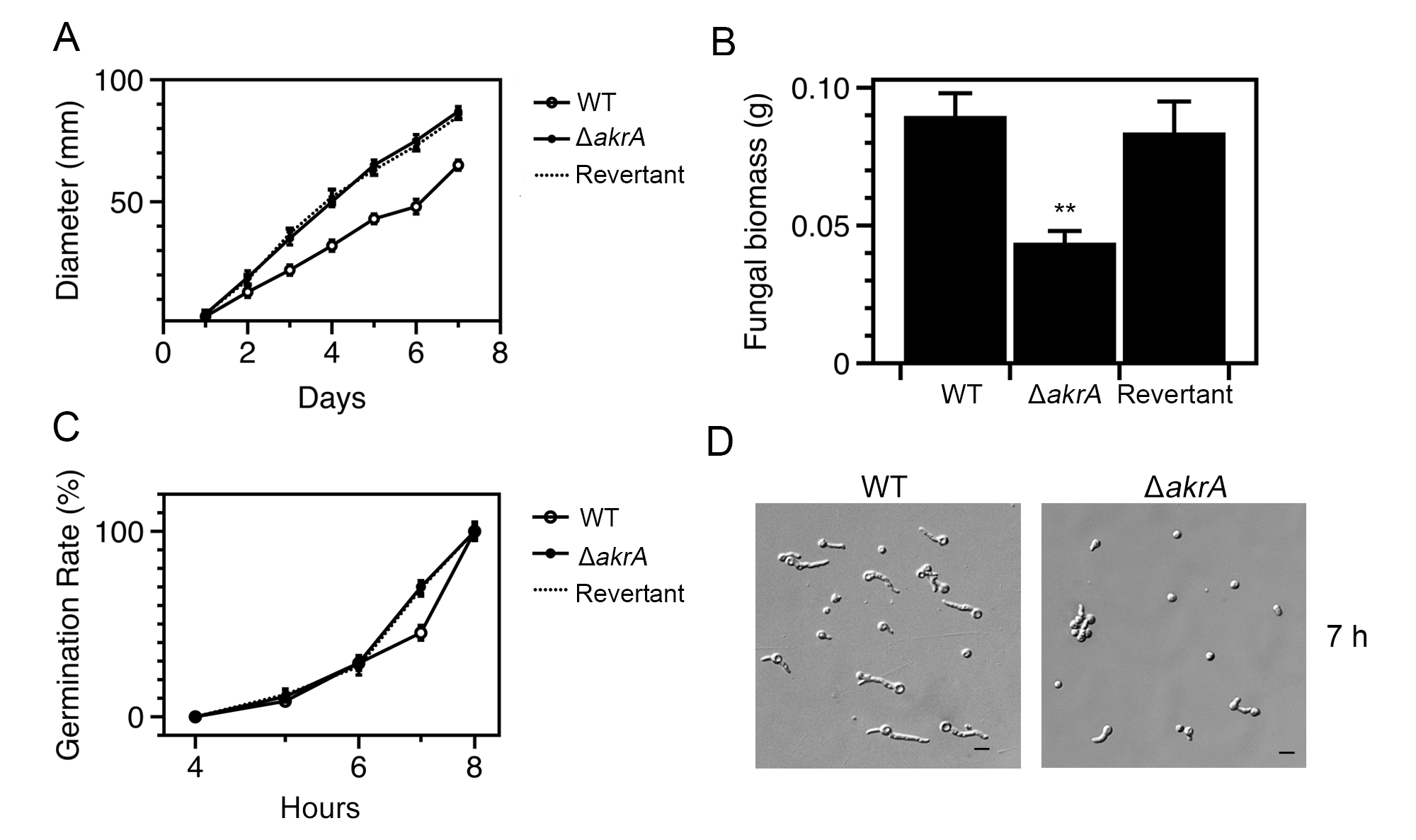

Supplement: S3 Fig — A. Comparison of mycelial extension rates of TN02A7 (WT), ΔakrA and revertant strains. Over 7 days, the colony diameter of each culture from spot-inoculated strains (each inoculum containing ~ 200 spores) was measured daily. B. Comparison of fungal biomass in liquid minimal medium after strains had been cultured for 24 h at 37°C, 220 rpm. Mycelia were dried and their dry weight was measured, **p<0.01. C. Germination rates in TN02A7 (WT), ΔakrA and revertant strains. Conidia were incubated in stationary liquid minimal media at the times indicated. 100 conidia for each strain were assessed for germination. These experiments were performed in triplicate, and the results are displayed as mean values with standard errors. D. Comparison of conidial germination of the TN02A7 (WT) and ΔakrA mutant imaged by differential interference contrast (DIC) microscopy after incubation for 7 h. (TIF) [file pgen.1005977.s003.tif]

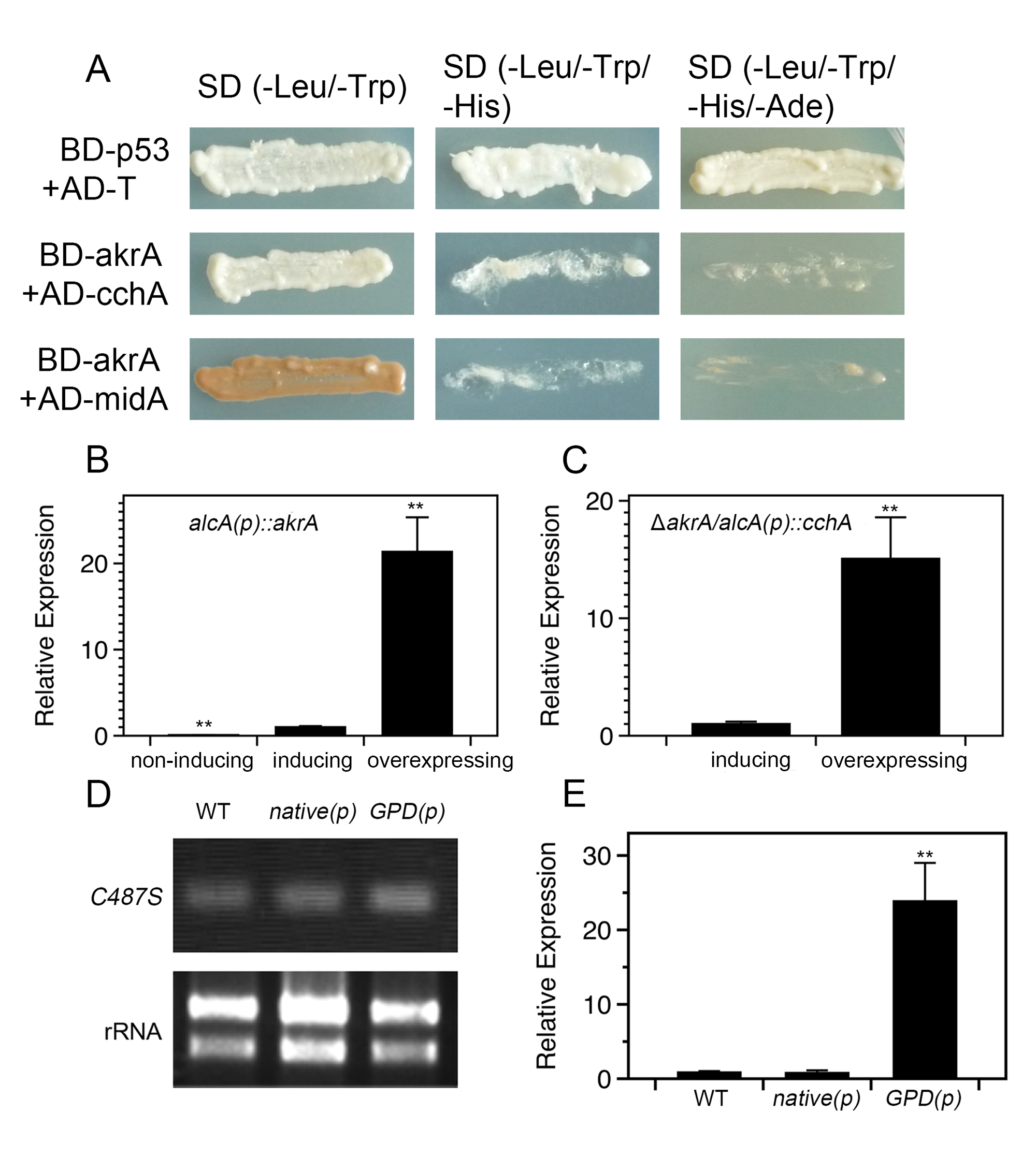

Supplement: S4 Fig — A. Physical interaction assay among AkrA, CchA and MidA revealed by yeast two-hybrid system. A cDNA fragment corresponding to the cytosol C-terminus of CchA and the full-length cDNA of MidA were placed in frame with the DNA activation domain of GAL4 in the pGADT7 while the full-length cDNA of AkrA was cloned to pGBKT7 vector. Protein–protein interactions were detected by growth in high-stringency media for selection (SD/-Ade/-His/-Leu/-Trp). pGADT7-T and pGBKT7-p53 were used together as a control for the interaction. B. Expression analysis by quantitative PCR of akrA using the alcA conditional promoter in liquid media (MMPDR, MMPGR and MMPGRT) providing non-inducing, inducing and overexpressing conditions, respectively. All mRNA levels were normalized to the mRNA level of actin (actA). The error bars indicate the standard deviation for three independent replicates, **p<0.01. C. Expression analysis of cchA using the alcA conditional promoter in liquid induced medium, overexpressed medium by quantitative PCR. All mRNA levels were normalized to an mRNA level of actin (actA). The error bars indicate the standard deviation for three independent replicates, **p<0.01. D, E. Expression of akrA using the akrA native or GPD promoter was examined using semi-quantitative PCR (D) and quantitative real-time PCR (E) from native(p)::akrAC487S and GDP(p)::akrAC487S strains. All mRNA levels were normalized to the mRNA level of actin (actA). The error bars indicate the standard deviation for three independent replicates, **p<0.01. (TIF) [file pgen.1005977.s004.tif]

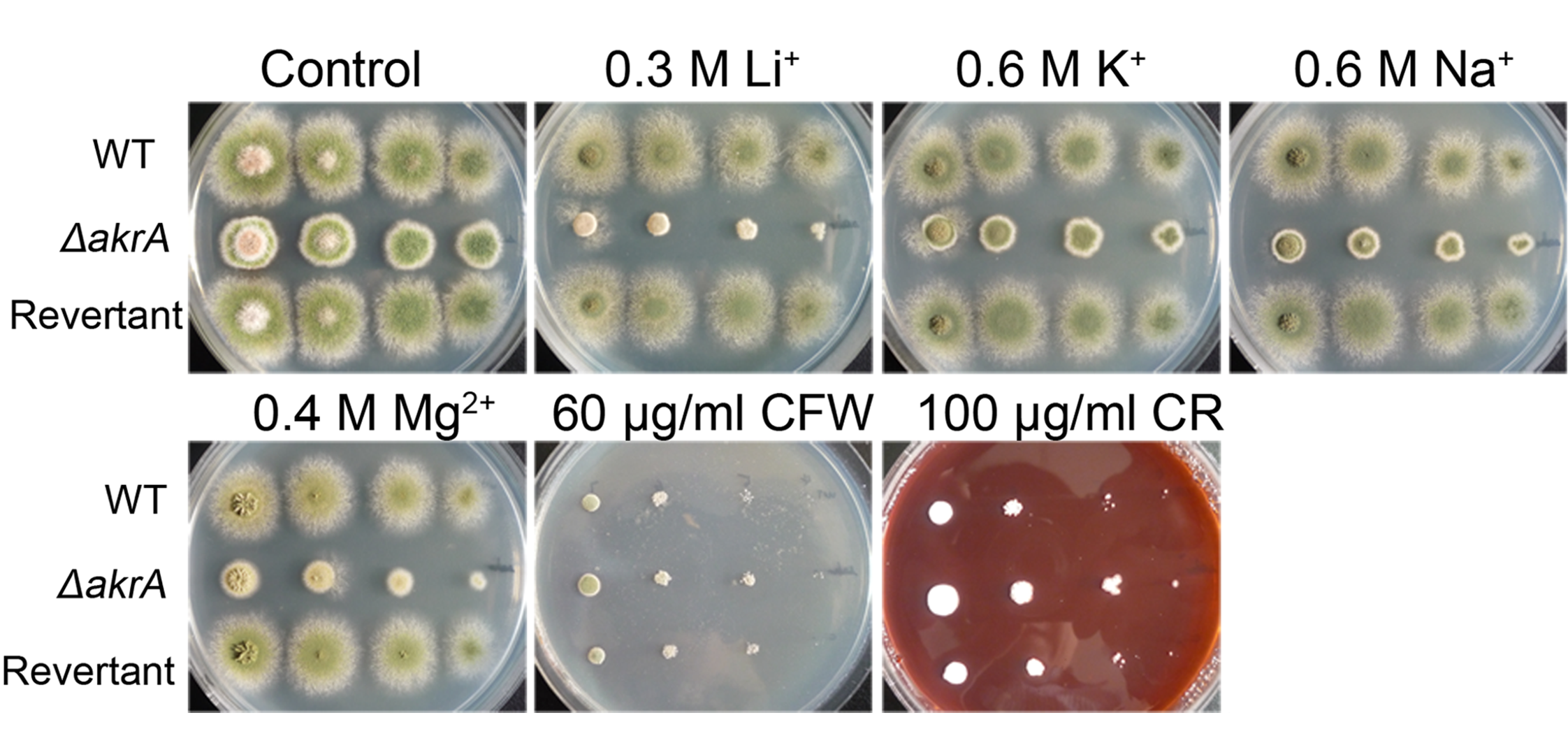

Supplement: S5 Fig — Phenotypic comparison of TN02A7 (WT), ΔakrA and revertant strains in minimal medium in the presence of the indicated cations and cell wall disrupting agents Congo Red and Calcofluor White. A series of 2 μL 10-fold dilutions derived from a starting suspension of 107 conidia/mL as indicated were spotted onto solid minimal medium supplemented with 0.3 M Li+, 0.6 M K+, 0.6 M Na+, 0.4 M Mg2+, 60 μg/mL Calcofluor White and 100 μg/mL Congo Red, respectively, and incubated at 37°C for 2.5 days. (TIF) [file pgen.1005977.s005.tif]

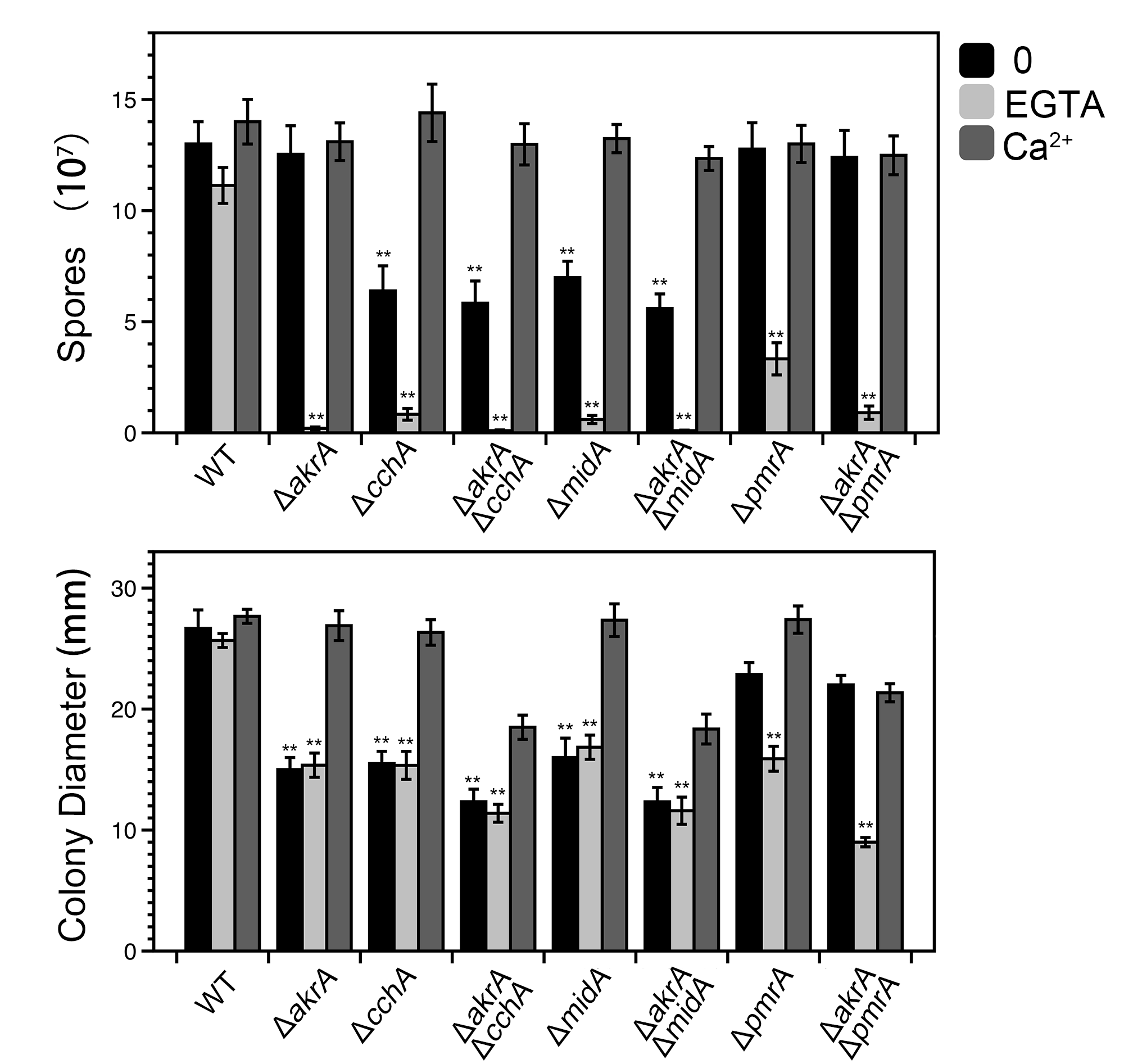

Supplement: S6 Fig — All the indicated strains were grown on the minimal medium in the presence of 1 mM EGTA or 20 mM CaCl2. Error bars represent standard deviation of three replicates, **p<0.01. (TIF) [file pgen.1005977.s006.tif]

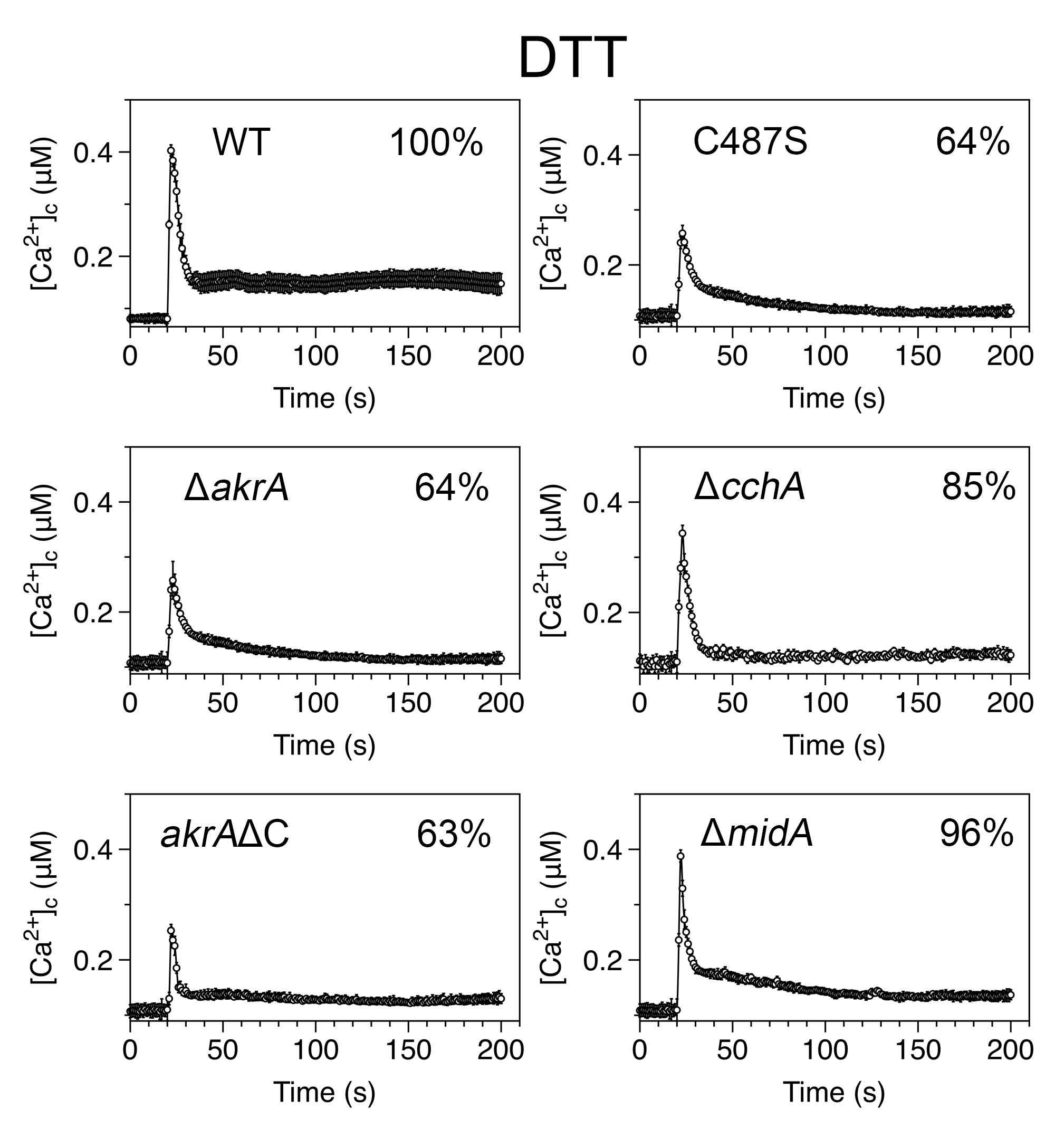

Supplement: S7 Fig — [Ca2+]c responses in the indicated strains to dithiothreitol (DTT) (10 mM). In each experiment, values represent averages of six wells and error bars represent SD (n = 6). (TIF) [file pgen.1005977.s007.tif]

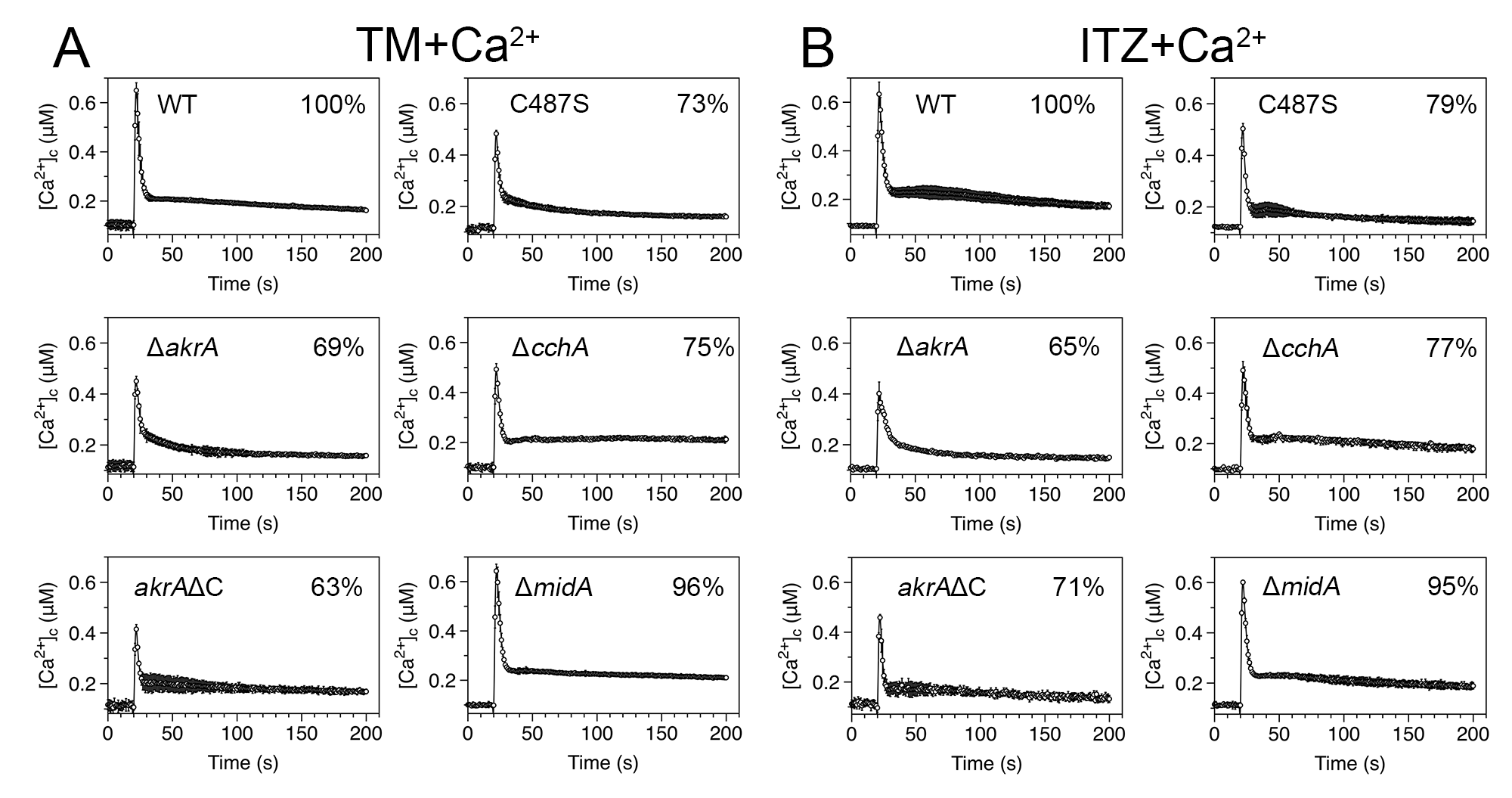

Supplement: S8 Fig — [Ca2+]c responses in the indicated strains to (A) tunicamycin (5 μg/mL) and (B) ITZ (1 μg/mL) supplemented with 5 mM CaCl2. Values represent averages of six well and error bars represent SD (n = 6). (TIF) [file pgen.1005977.s008.tif]

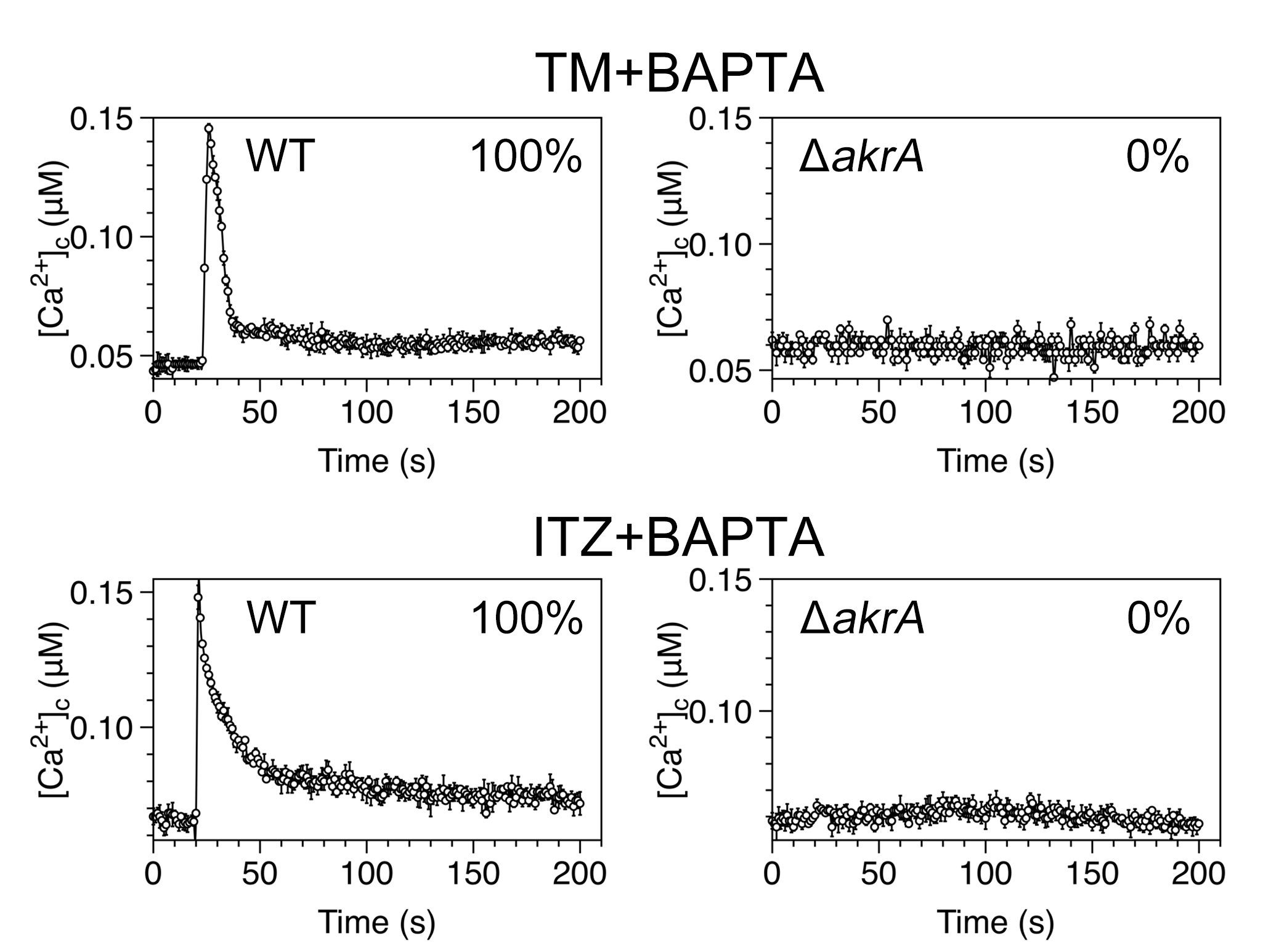

Supplement: S9 Fig — The effect of pretreatment with the calcium chelator BAPTA (8 mM) on the [Ca2+]c transient induced by tunicamycin (5 μg/mL) and itraconazole (1 μg/mL) is shown. In each experiment, values represent averages of six well and error bars represent SD (n = 6). (TIF) [file pgen.1005977.s009.tif]

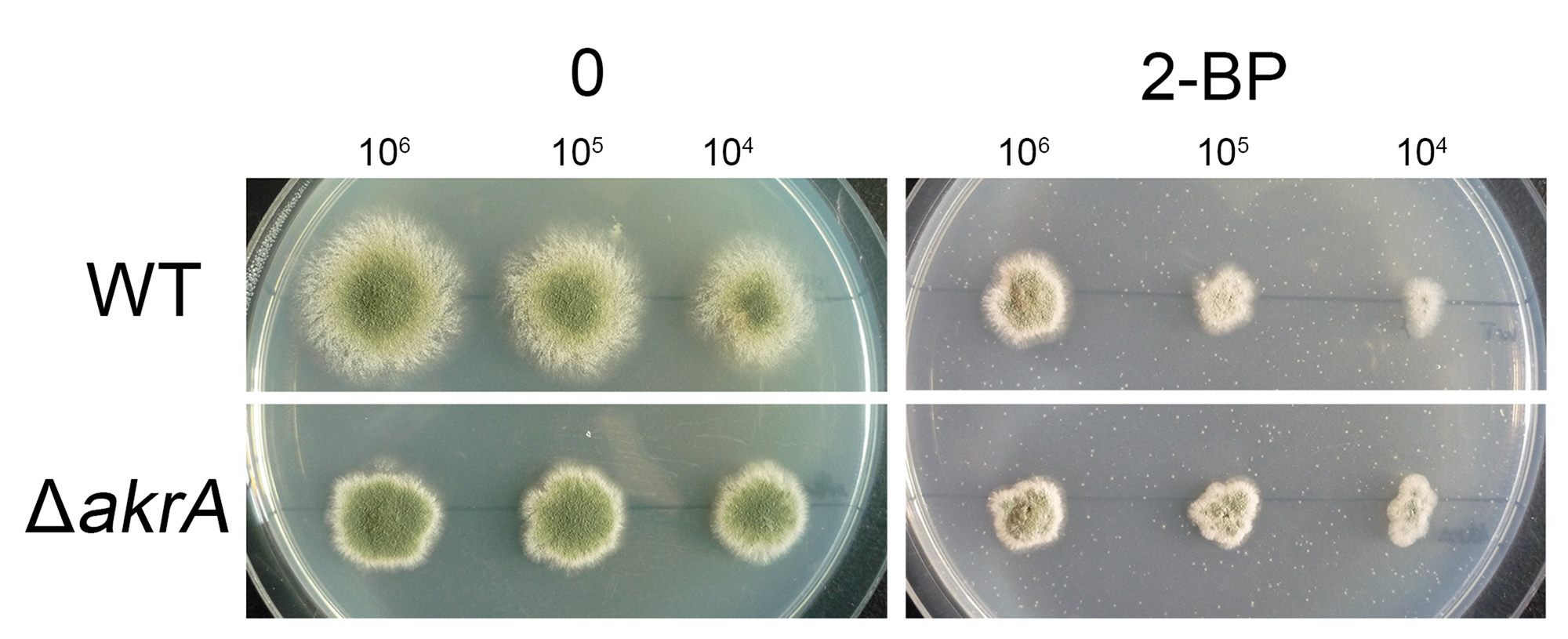

Supplement: S10 Fig — The colony morphology of TN02A7 (WT) and ΔakrA strains in a series of 2 μL 10-fold dilutions derived from a starting suspension of 106 conidia/mL grown on minimal medium at 37°C for 2.5 days in the presence or absence of 100 μM 2-bromopalmitate (2-BP). (TIF) [file pgen.1005977.s010.tif]
