# Supplementary material for: Palmitoylation of the Cysteine Residue in the DHHC Motif of a Palmitoyl Transferase Mediates Ca2+ Homeostasis in Aspergillus
Source: PLoS Genet. 2016 Apr 8;12(4):e1005977. doi: 10.1371/journal.pgen.1005977 (PMC4825924; doi:10.1371/journal.pgen.1005977)
Supplement: S1 Table — (DOCX) [file pgen.1005977.s011.docx]

**S1 Table. Strains used in this study**

| **Strain name** | **Deleted and labeled gene name** | **Genotype** | **Source** |
| --- | --- | --- | --- |
| **TN02A7** | **Parental strain** | ***pyrG89; pyroA4, nkuA::argB2; riboB2*** | **FGSC** |
| **ZYA01** | **Δ*cnaA*** | ***ΔcnaA::pyroA; pyroA4, pyrG89*** | **This work** |
| **ZYA02** | **Δ*akrA*** | ***ΔakrA::pyrG; pyrG89; pyroA4, nkuA::argB2; riboB2*** | **This work** |
| **ZYA03** | **Δ*pmrA*** | ***ΔpmrA::pyrG; pyrG89; pyroA4,nkuA::argB2; riboB2*** | **This work** |
| **ZYA04** | **Δ*akrA*Δ*cnaA*** | ***ΔcnaA::pyroA; pyroA4; ΔakrA::pyrG; pyrG89*** | **This work** |
| **ZYA05** | ***akrA*-recon** | ***ΔakrA::pyrG; pyrG89; nkuA::argB2; riboB2; native(p)::akrA*** | **This work** |
| **ZYA06** | **Δ*akrA*Δ*cchA*** | ***ΔakrA::pyrG; pyroA4; nkuA::argB2; ΔcchA::pyrG; pyrG89; riboB2*** | **This work** |
| **ZYA07** | **Δ*akrA*Δ*midA*** | ***ΔakrA::pyrG; pyroA4; nkuA::argB2; ΔmidA::pyrG; pyrG89; riboB2*** | **This work** |
| **ZYA08** | **Δ*akrA*Δ*pmrA*** | ***ΔakrA::pyrG; pyroA4; nkuA::argB2; ΔpmrA::pyrG; pyrG89*** | **This work** |
| **ZYA09** | ***alcA(p)*::GFP-*akrA*** | ***pyrG89; pyroA4, nkuA::argB2; alcA(p)::GFP-akrA::pyr-4; riboB2*** | **This work** |
| **ZYA10** | ***alcA(p)*::GFP-*akrA*^C487S^** | ***pyrG89; nkuA::argB2; alcA(p)::GFP-akrA^C487^::pyr-4; riboB2*** | **This work** |
| **ZYA11** | ***alcA(p)*::GFP-*cchA*** | ***pyrG89; pyroA4,nkuA::argB2; alcA(p)::GFP-cchA::pyr-4; riboB2*** | **This work** |
| **ZYA12** | ***ΔakrA alcA(p)*::GFP-*cchA*** | ***ΔakrA::pyrG; pyrG89; pyroA4, nkuA::argB2; alcA(p)::GFP-cchA::pyr-4*** | **This work** |
| **ZYA13** | ***alcA(p)*::GFP-*akrA* MAD2013** | ***pyrG89; pyroA4,nkuA::argB2; alcA(p)::GFP-akrA::pyr-4; riboB2; pyroA-gpdA^mini^::mrfp:PH^OSBP^*** | **This work** |
| **ZYA14** | ***ΔakrA alcA(p)*::GFP-*pmrA*** | ***ΔakrA::pyrG; pyrG89; pyroA4,nkuA::argB2; alcA(p)::GFP-pmrA::pyr-4*** | **This work** |
| **ZYA15** | ***akrA*ΔC** | ***akrAΔC::pyrG; nkuA::argB2; pyroA;pyrG89; riboB2*** | **This work** |
| **ZYA16** | ***native(p)::akrA*^C487S^** | ***ΔakrA::pyrG; pyrG89; nkuA::argB2; riboB2; native(p)::akrA^C487S^*** | **This work** |
| **ZYA17** | ***GPD(p)::akrA*^C487S^** | ***ΔakrA::pyrG;pyrG89; nkuA::argB2; riboB2; GPD(p)::akrA C487S*** | **This work** |
| **ZYA18** | **TN02A7-AEQ** | ***pyrG89; nkuA::argB2; riboB2; pAEQ-aeqS*** | **This work** |
| **ZYA19** | **Δ*akrA*-AEQ** | ***ΔakrA::pyrG; pyrG89; nkuA::argB2; riboB2; pAEQ-aeqS*** | **This work** |
| **ZYA20** | **Δ*midA*-AEQ** | ***ΔmidA::pyrG; pyrG89; nkuA::argB2; riboB2; pAEQ-aeqS*** | **This work** |
| **ZYA21** | **Δ*cchA*-AEQ** | ***ΔcchA::pyrG; pyrG89; nkuA::argB2; riboB2; pAEQ-aeqS*** | **This work** |
| **ZYA22** | **Δ*pmrA*-AEQ** | ***ΔpmrA::pyrG; pyrG89; nkuA::argB2; riboB2; pAEQ-aeqS*** | **This work** |
| **ZYA23** | **Δ*akrAΔcchA*-AEQ** | ***ΔakrA::pyrG; nkuA::argB2; ΔcchA::pyrG; pyrG89; riboB2; pAEQ-aeqS*** | **This work** |
| **ZYA24** | **Δ*akrAΔmidA*-AEQ** | ***ΔakrA::pyrG; nkuA::argB2; ΔmidA::pyrG; pyrG89; riboB2; pAEQ-aeqS*** | **This work** |
| **ZYA25** | **Δ*akrAΔpmrA*-AEQ** | ***ΔakrA::pyrG; nkuA::argB2; ΔpmrA::pyrG; pyrG89; pAEQ-aeqS*** | **This work** |
| **ZYA26** | ***akrA*-recon-AEQ** | ***ΔakrA::pyrG; pyrG89; nkuA::argB2; native(p)::akrA; pAEQ-aeqS*** | **This work** |
| **ZYA27** | ***native(p) akrA*^C487S^-AEQ** | ***ΔakrA::pyrG; pyrG89; nkuA::argB2; native(p)::akrA^C487S^; pAEQ-aeqS*** | **This work** |
| **ZYA28** | **Flag-AkrA** | ***ΔakrA::pyrG; pyrG89; nkuA::argB2; riboB2; N-Flag tagged-native(p)::akrA*** | **This work** |
| **ZYA29** | **Flag-AkrA^C487S^** | ***ΔakrA::pyrG ;pyrG89; nkuA::argB2; riboB2; N-Flag tagged-native(p)::akrA^C487S^*** | **This work** |
| **ZYA30** | **Δ*Afakr*** | ***ΔKU80; pyrG;* Δ*Afakr::pyrG*** | **This work** |
