# Supplementary material for: Palmitoylation of the Cysteine Residue in the DHHC Motif of a Palmitoyl Transferase Mediates Ca2+ Homeostasis in Aspergillus
Source: PLoS Genet. 2016 Apr 8;12(4):e1005977. doi: 10.1371/journal.pgen.1005977 (PMC4825924; doi:10.1371/journal.pgen.1005977)
Supplement: S2 Table — (DOCX) [file pgen.1005977.s012.docx]

**S2 Table.** **Primers used in this study**

| **Name** | **Sequence (5’ to 3’)** | **Purpose** |
| --- | --- | --- |
| akrA-P1 | CTGCGTGTAGCGATGAAGAC | Fusion PCR for *akrA* knockout |
| akrA-P2 | AGAACTCACGCGACTGGAGA |  |
| akrA-P3 | GAGGGTGAAGAGCATTGTTTGAGGCCCTCCGTTTTCTAGCCTCGTAGGGA |  |
| akrA-P4 | CGCATCAGTGCCTCCTCTCAGACAGAGCAAGCAACTGTGGCGAGGCACGA |  |
| akrA-P5 | TTTCTTGCACCGTAGTCTGG |  |
| akrA-P6 | CTGGCGGAGCAGCGAATGAC |  |
| K1 | TCTTCCGGGGATCCTCCATC | *akrA* knockout confirmation |
| K2 | CTAAACGATGTCTGCCGCTTCA |  |
| K3 | CTGCGTGTAGCGATGAAGAC |  |
| K4 | TAGGGACCGAGACCTGTATC |  |
| akrA-trunc-P1 | CTCAGCGGTGCCACTATTA | Fusion PCR for AkrA C terminal deletion |
| akrA-trunc-P2 | CTTGTCTTGCTCCTTCACCA |  |
| akrA-trunc-P3 | CTCTAGATGCATGCTCGAGCTGCTGCCAAGTTTTGGCTA |  |
| akrA-trunc-P4 | CAGTGCCTCCTCTCAGACAGGCGAAATTGTCAACTATAACCG |  |
| akrA-trunc-P5 | CTACGGAGTACAGCCTTAACCT |  |
| akrA-trunc-P6 | ATCAAAGCCATCACCTCCAC |  |
| alc-akrA-5’ | ATATAGCGGCCGCTGTCTTCCGGGGATCCTC | *alc(p)::GFP-akrA* conditional strain |
| alc-akrA-3’ | GAATGTCTAGATGACTGGCAGCCTTCTTGG |  |
| G1 | CTGCGTGTAGCGATGAAGAC | *alc(p)::GFP-akrA* conditional strain confirmation |
| G2 | TGACTGGCAGCCTTCTTGG |  |
| G3 | GACACCCTCGTCAACAGGATCG |  |
| G4 | TGACTGGCAGCCTTCTTGG |  |
| primer A | GTCCAGCGGCGAGAATAC | Site-directed mutagenesis |
| primer B | CCAGGGGGAGTGACTGTG |  |
| primer C | CACAGTCACTCCCCCTGG |  |
| primer D | GGCTGGTGGATTGGAGGT |  |
| GPD-5’ | GAATTCCCTTGTATCTCTACACA | *GPD(p)::akrA* C487S construction |
| GPD-3’ | GGAAATAAAGGTTCTTGGATG |  |
| akrA-GPD-5’ | CATCCAAGAACCTTTATTTCCATGTCTTCCGGGGATCCT |  |
| Anpyro-5' | TTGGCGGGTAAGTCAGATAATAG |  |
| Anpyro-3' | CTGACTTGACGCTTTCTCTT |  |
| Afpyrg-5’ | GCCTCAAACAATGCTCTTCACC |  |
| Afpyrg-3’ | CTGTCTGAGAGGAGGCACTGATG |  |
| AD-akrA-5’ | GGGAATTCCATATGTCTTCCGGGGATCCTCCATC | Yeast two hybrid |
| AD-akrA-3’ | CCATCGATCTAAACGATGTCTGCCGCTTCA |  |
| BD-akrA-5’ | GGGAATTCCATATGTCTTCCGGGGATCCTCCATC |  |
| BD-akrA-3’ | ACGGCGTCGACCTAAACGATGTCTGCCGCTTCA |  |
| AD-cchA-5’ | TCCCCCGGGGCTGCTTTTTCCGGACGAGTT |  |
| AD-cchA-3’ | CGCGGATCCTTATGTCTCGTCCCTTGGTCG |  |
| AD-midA-5’ | CGGAATTCATGCAACGGCAAAACGC |  |
| AD-midA-3’ | TCCCCCGGGCGCTAAAACACCATCACAAT |  |
| N-3XFLAG-5’ | CTACGAGGCTAGAAAACGGAGGATGGACTATAAGGACCACGACGG | FLAG-tag |
| N-3XFLAG-3’ | CCAGATGGAGGATCCCCGGAAGACTTATCGTCATCGTCTTTGTAATCA |  |
| pyro-cre-5’ | GACTAGTCCTGCAGGTTTATTCTCCTGCTTGCTCC | *alcA(p)::GFP-akrA C487S* construction |
| pyro-cre-3’ | AAGGGCAATTCGTTTTCTTGGCTCTATCGTATTCTT |  |
| alc-up | CTGAAAAGCTGATTGTGATAGTTCCCACTTGTCCGTC |  |
| new primer D | ACCTCCAATCCACCAGCC |  |
| GPD-N-3XFLAG-5’ | TATATTCATCTTCCCATCCAAGAACCTTTATTTCCGACTATAAGGACCACGACGG |  |
| GPD-N-3XFLAG-3’ | AGATGGAGGATCCCCGGAAGACATCTTATCGTCATCGTCTTTGTAATCA |  |
| RT-akrA-5’ | CCAAGAGGCTGCCAGTCAA | qRT-PCR *for akrA* |
| RT-akrA-3’ | ATACCCAGGATCTGCGACCA |  |
| RT-cchA-5’ | TTCCACTCCGTCATAATCG | qRT-PCR for *cchA* |
| RT-cchA-3’ | GCTCATAAACTCGCCAACAC |  |
| RT-actA-5’ | TCTTCCAGCCCAGCGTTCT | qRT-PCR for *actinA* |
| RT-actA-3’ | GGGCGGTGATTTCCTTCTG |  |
